# Supplementary material for: Nanoproteomics enables proteoform-resolved analysis of low-abundance proteins in human serum
Source: Nat Commun. 2020 Aug 6;11:3903. doi: 10.1038/s41467-020-17643-1 (PMC7411019; doi:10.1038/s41467-020-17643-1)
Supplement: Supplementary file 1 — Supplementary Information [file 41467_2020_17643_MOESM1_ESM.pdf]

Supplementary Information

Nanoproteomics enables proteoform-resolved analysis of low-abundance proteins in human serum

Timothy N. Tiambeng[1,†], David S. Roberts[1,†], Kyle A. Brown[1], Yanlong Zhu[2,3], Bifan Chen[1], Zhijie Wu[1], Stanford D. Mitchell[3,4], Tania M. Guardado-Alvarez[1], Song Jin[1]*, Ying Ge[1,2,3]*

[1]Department of Chemistry, [2]Human Proteomics Program, School of Medicine and Public Health, [3]Department of Cell and Regenerative Biology, [4]Molecular and Cellular Pharmacology Training Program, University of Wisconsin–Madison, Wisconsin 53719, USA.

[†]These authors contributed equally to this work.

*Correspondence should be addressed to S.J. (jin@chem.wisc.edu) and Y.G. (ying.ge@wisc.edu).

Table of Contents **Page**

Supplementary Figures 1-31	2-32
Supplementary Tables 1-8	33-40

Supplementary Figures

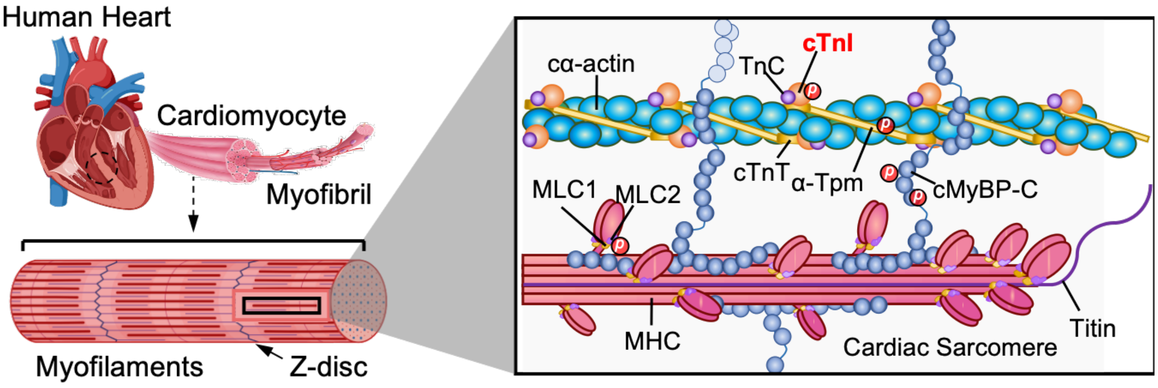

Supplementary Figure 1
Schematic molecular composition of the cardiac sarcomere with the location of cardiac troponin I (cTnI) highlighted in Red**.** Cardiomyocytes are arranged into tubular structures called myofibrils and further sub-organized into cardiac sarcomeres, the smallest contractile units in the myofibril. Sarcomeres are composed of myofilaments (thin and thick filaments) flanked by dense protein structures called Z-discs. Myofilaments include thin filament proteins [cTnI (cardiac troponin I), TnC (troponin C), cTnT (cardiac troponin T), cα-actin (cardiac alpha actin), and α-Tpm (alpha-tropomyosin)] and thick filament proteins [MHC (myosin heavy chain), MLC1 (essential light chain), MLC2 (regulatory light chain), cMyBP-C (cardiac myosin binding protein C), and titin]. p denotes protein phosphorylation.

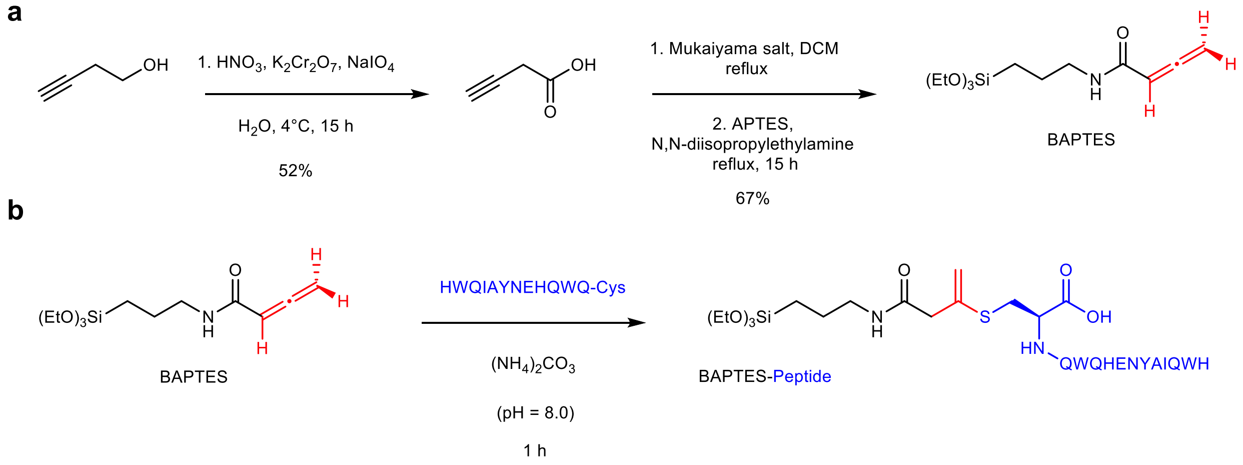

Supplementary Figure 2

Synthesis of N-(3-(triethoxysilyl)propyl)buta-2,3-dienamide (BAPTES) organosilane monomer and peptide modification with BAPTES.

a, Scheme illustrating the facile synthesis of the BAPTES organosilane monomer used to silanize the 8 nm superparamagnetic Fe_3O_4 NPs. **b**, Cysteine modification of the high affinity cTnI-binding peptide with BAPTES monomer, illustrating potential functionalization of BAPTES surface-silanized NPs with cTnI-binding peptide. Yields are reported as the average of three independent synthesis experiments.

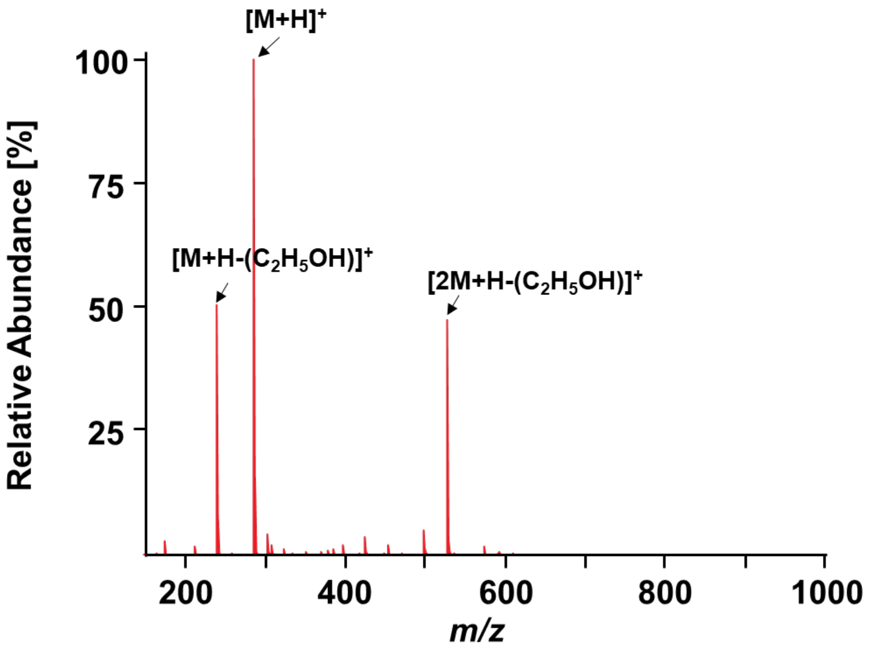

Supplementary Figure 3
High resolution ESI-MS of BAPTES monomer. ESI-MS for $C_{13}H_{25}NO_4Si$ $[M+H]^+$ observed: 288.162 *m/z*, $[M+H]^+$ calculated: 288.162 *m/z*. Neutral mass losses are denoted on spectra: $[M+H-C_2H_5OH]^+$ observed: 242.120 *m/z,* $[2M+H-C_2H_5OH]^+$ observed: 529.276 *m/z.*

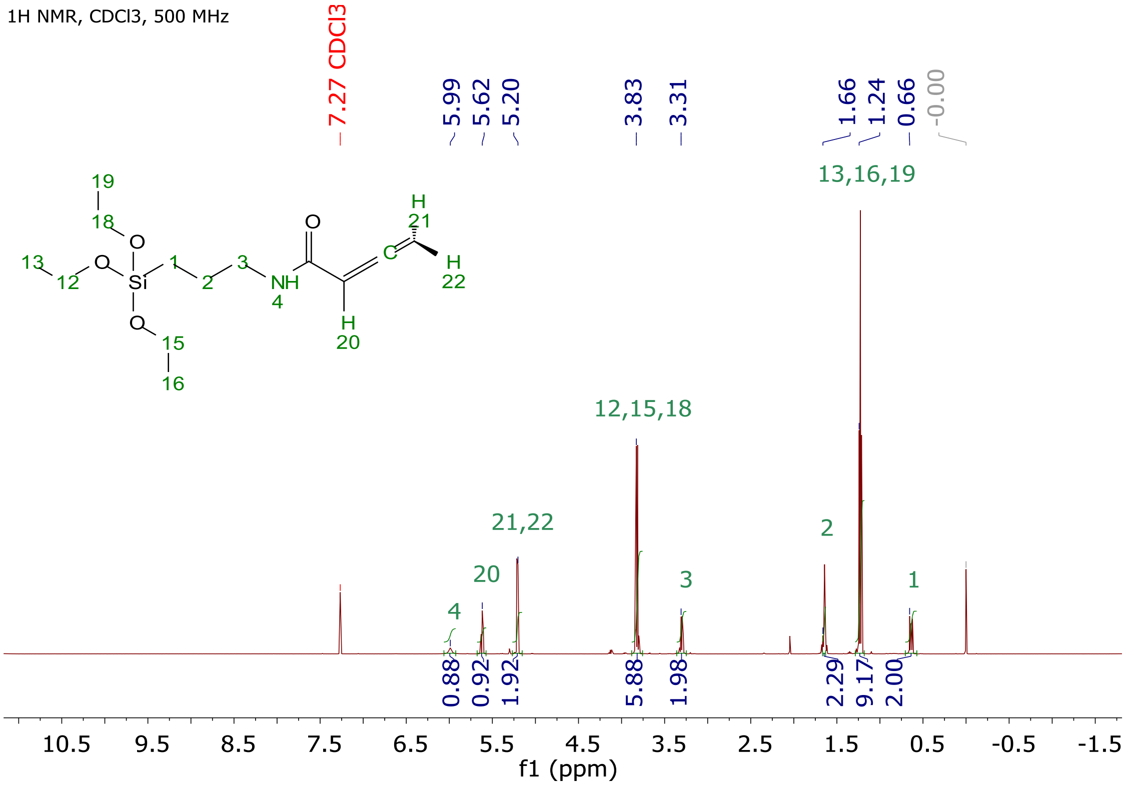

Supplementary Figure 4
^{1}H NMR of BAPTES monomer.
^{1}H NMR (500 MHz, Chloroform-*d*) δ (ppm) 5.99 (s, 1H), 5.62 (t, J = 6.6 Hz, 1H), 5.20 (d, J = 6.7 Hz, 2H), 3.83 (q, J = 7.0 Hz, 6H), 3.31 (q, J = 6.7 Hz, 2H), 1.66 (m, 2H), 1.24 (t, J = 7.0 Hz, 9H), 0.66 (m, 2H). ^{1}H integrations confirm isolation and synthesis of BAPTES monomer.

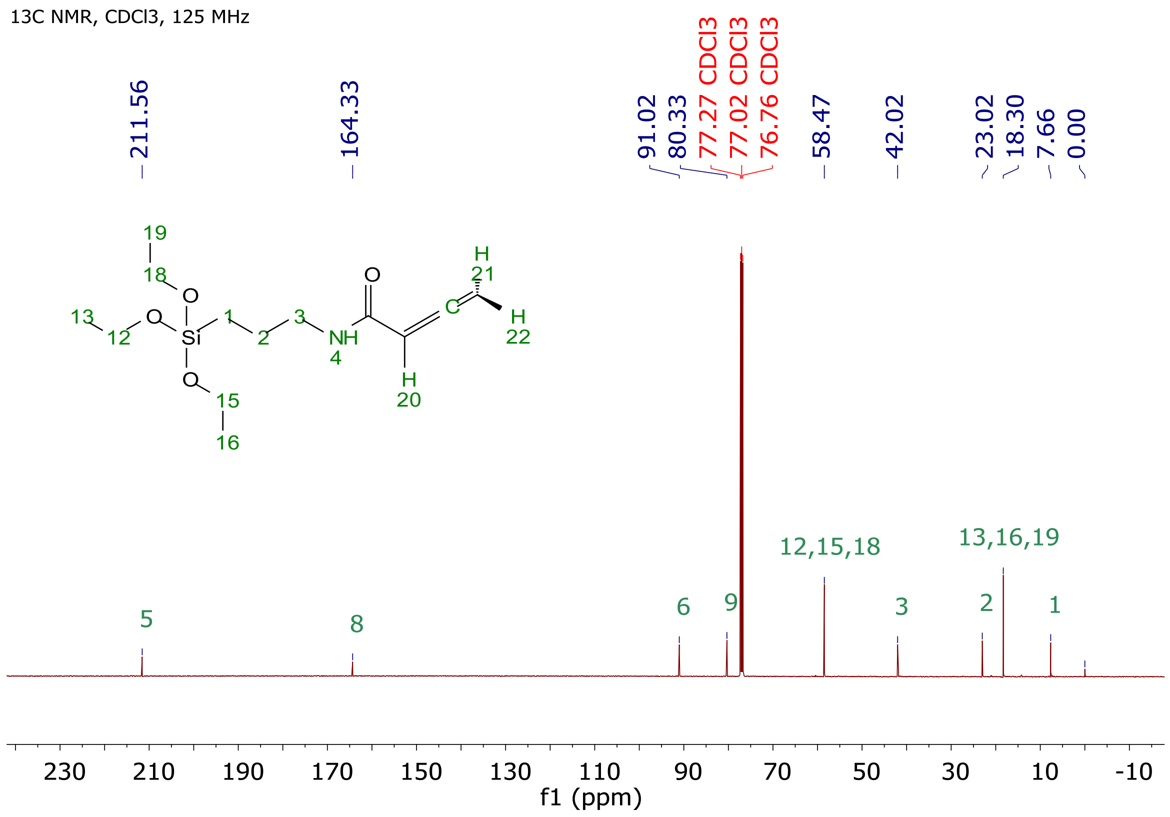

Supplementary Figure 5
^{13}C NMR of BAPTES monomer.
^{13}C NMR (125 MHz, Chloroform-*d*) δ (ppm) 211.56, 164.33, 91.02, 80.33, 58.47, 42.02, 23.02, 18.30, 7.66. ^{13}C NMR confirms the synthesis of the BAPTES monomer and the presence of 9 unique ^{13}C chemical environments.

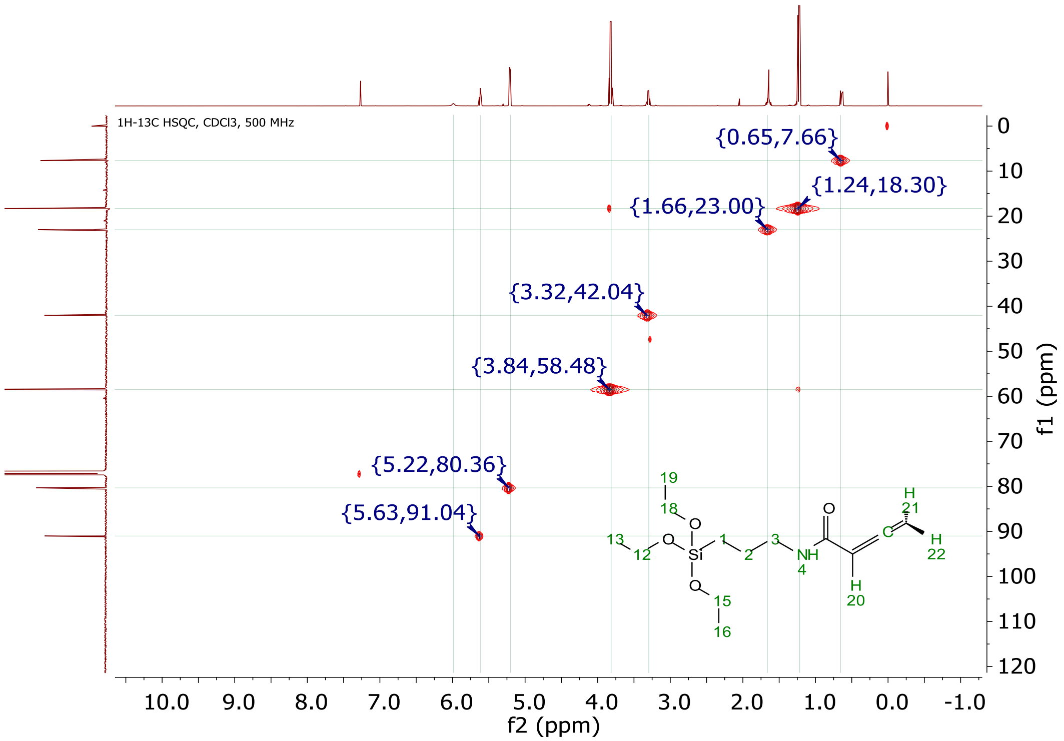

Supplementary Figure 6

^{1}H-^{13}C Heteronuclear Single Quantum Coherence (HSQC) NMR of BAPTES monomer.

^{1}H-^{13}C HSQC (500 MHz, Chloroform-*d*) correlates the observed ^{1}H and ^{13}C signals present in the BAPTES molecule, confirming purification and synthesis of BAPTES monomer.

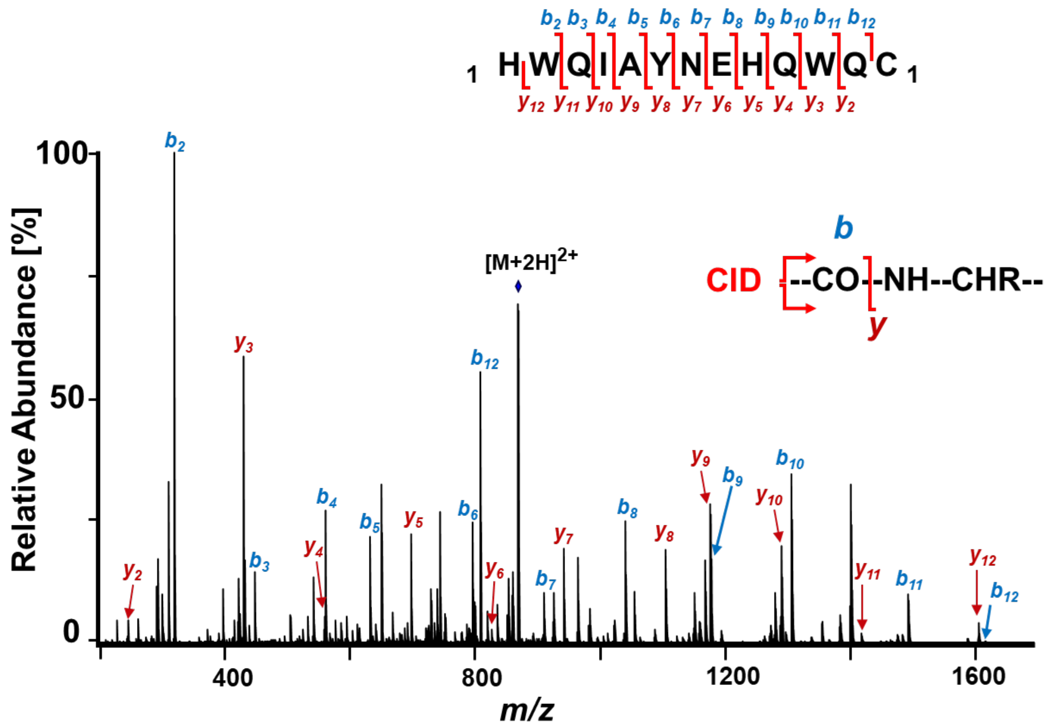

Supplementary Figure 7

ESI-MS of cTnI affinity peptide showing full sequence coverage.

The sequence of the cTnI affinity peptide was validated by tandem MS (MS/MS) with full sequence coverage, using a 12 T Bruker Fourier transform ion cyclotron resonance (FTICR) mass spectrometer. A mass accuracy cut-off of 10 ppm was used for MS/MS fragment ion assignments. All ion identifications were manually validated. ESI-MS for $C_{79}H_{103}N_{23}O_{21}S_1$ $[M+2H]^{2+}$ observed: 871.884 *m/z*, $[M+2H]^{2+}$ calculated: 871.878 *m/z*.

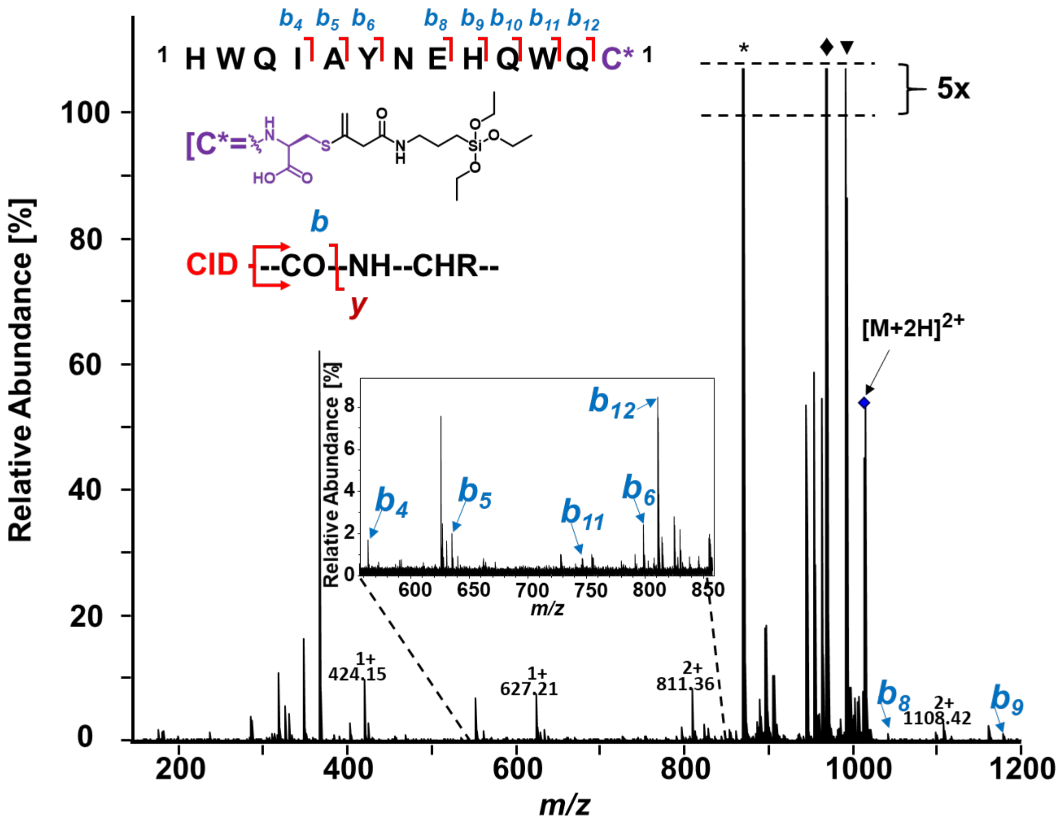

Supplementary Figure 8

ESI-MS of cTnI affinity peptide coupled to BAPTES monomer showing site-specific addition of the peptide to the allene carboxamide functional handle.

The sequence of the BAPTES ($C_{13}H_{25}NO_4Si$)-modified cTnI affinity peptide was validated by tandem MS (MS/MS) sequence coverage using a 12 T Bruker Fourier transform ion cyclotron resonance (FTICR) mass spectrometer. Representative ions are denoted in the spectrum, with the inset showing an expansion of the 550-850 *m/z* range. A mass accuracy cut-off of 10 ppm was used for MS/MS fragment ion assignments. All ion identifications were manually validated. ESI-MS for $C_{92}H_{128}N_{24}O_{25}SiS$: $[M+2H]^{2+}$ observed: 1015.451 *m/z*, $[M+2H]^{2+}$ calculated: 1015.455 *m/z*. Neutral mass losses are denoted on spectra: Solid triangle (▼), $[M+2H-C_2H_5OH]^{2+}$; Solid diamond (♦), $[M+2H-2(C_2H_5OH)]^{2+}$; Asterisk (*), $[M+2H-C_{13}H_{25}NO_4Si]^{2+}$.

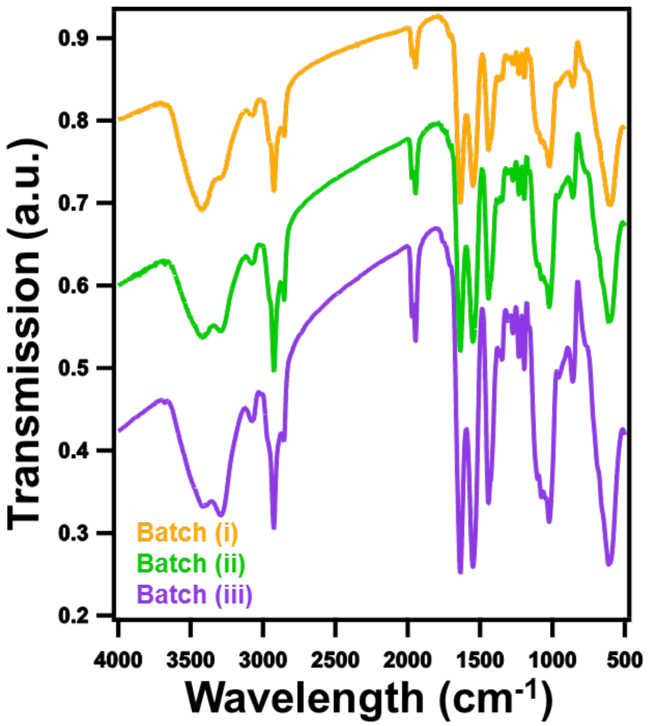

Supplementary Figure 9

Inter-batch reproducibility of surface functionalized NPs confirmed by Fourier transform infrared (FTIR) analysis.

FTIR traces of three representative batches (i-iii) of Fe_3O_4-BAPTES NPs synthesized under the same reaction conditions. Traces are offset along the *y*-axis for clarity.

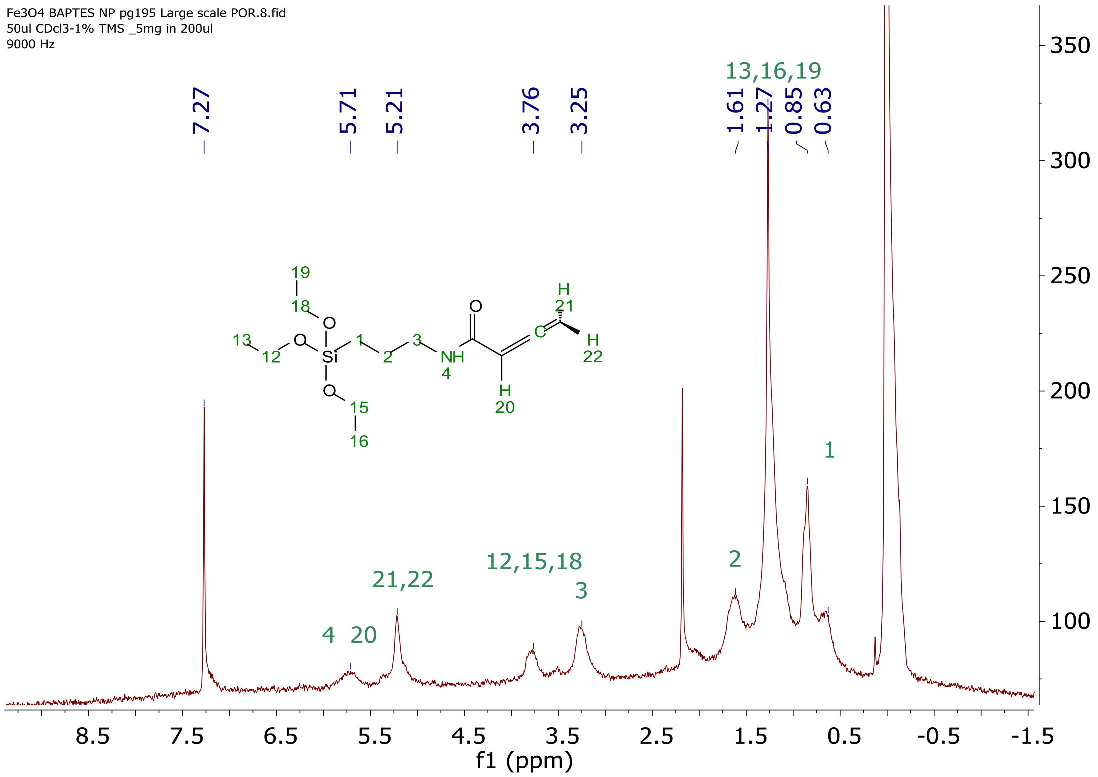

Supplementary Figure 10
High resolution magic angle spinning nuclear magnetic spectroscopy (HRMAS) analysis of silanized NPs.
^{1}H NMR spectrum of Fe_3O_4-BAPTES NPs performed in Chloroform-*d* at a 9 kHz magic angle spinning (MAS) rate. δ (ppm) 5.71, 5.21, 3.76, 3.25, 1.61, 1.27, 0.85, 0.63. Characteristic allene carboxamide signatures belonging to the BAPTES monomer indicate the successful surface silanization of the starting Fe_3O_4-OA NPs to the Fe_3O_4-BAPTES NPs (i.e., unfunctionalized NPs, later referred to as NP-Ctrl).

a

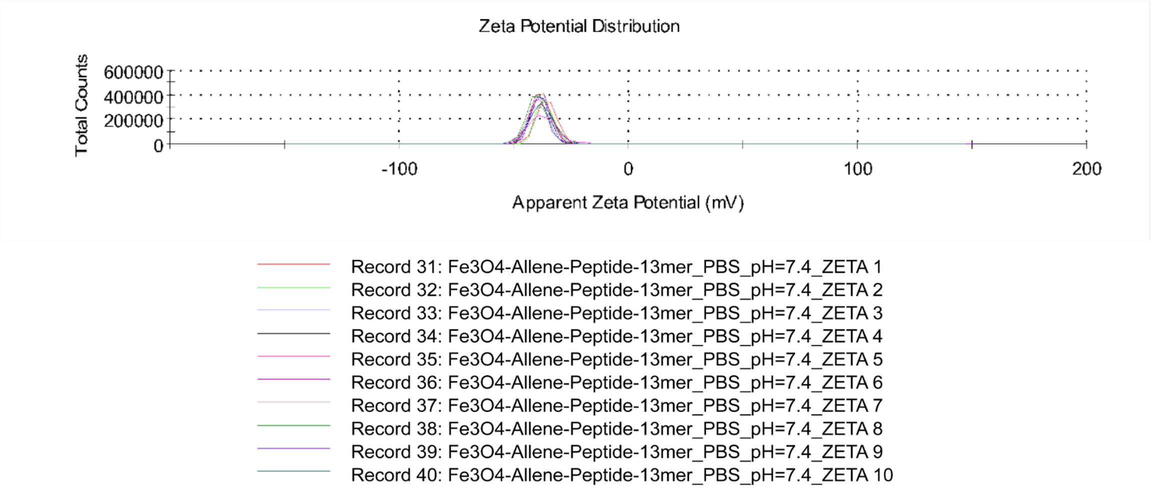

b

	Electrophoretic Mobility (μmcm/Vs)	Conductivity (mS/cm)	Zeta Potential (mV)
**Average**	-3.01	0.30	-38.2
**Standard Deviation**	0.10	0.0044	1.2
**Relative Standard Deviation (%)**	3.3	1.4	3.2

Supplementary Figure 11
Summary of zeta potential measurements on NP-Pep.
a, Plots of apparent zeta potentials for NP-Pep suspended in 0.1x PBS buffer (pH = 7.4) at a mass loading concentration of 0.25 mg/mL. Data are representative of n = 10 independent NP-Pep syntheses from the same NP-BAPTES batch. **b**, Table summary of electrophoretic mobility, conductivity, and zeta potential results as shown in (**a**).

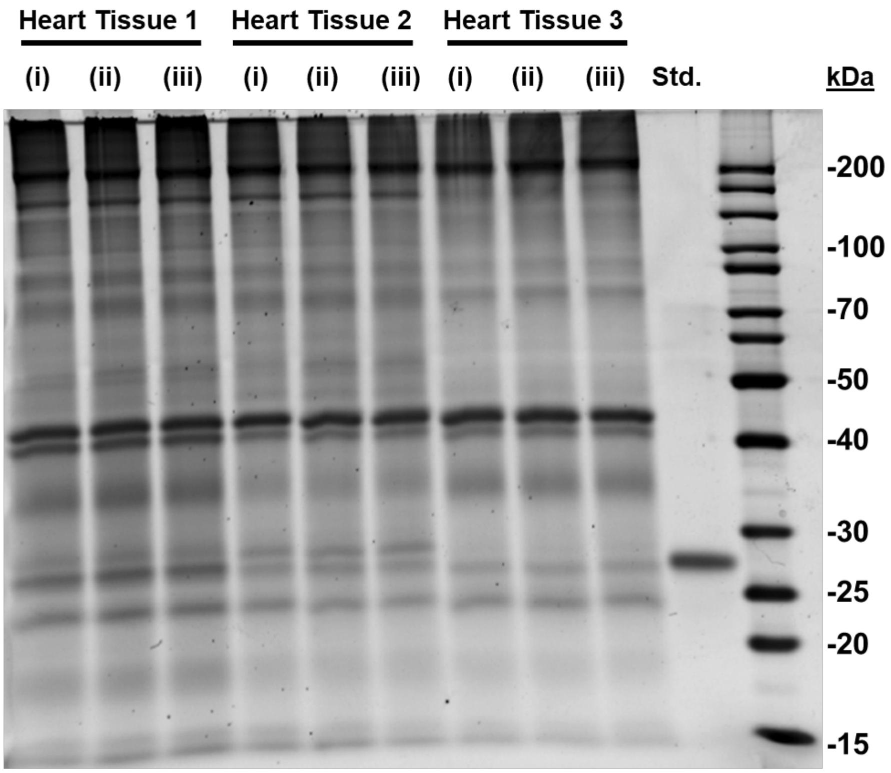

Supplementary Figure 12
Reproducibility of sarcomeric protein extractions from heart tissue.
SDS-PAGE stained with SYPRO Ruby visualizing sarcomeric proteins obtained from the heart tissue protein extraction procedure. The heart tissue protein extraction procedure is listed in Supplementary Table 4. Three independent protein extractions (i-iii) were performed on three different heart tissue samples (1, donor tissue; 2, ischemic tissue; 3, post-mortem tissue). Equal protein amount (1 µg) was loaded in each lane, demonstrating the reproducibility of protein extractions within three biological samples. Std., human cTnI protein standard.

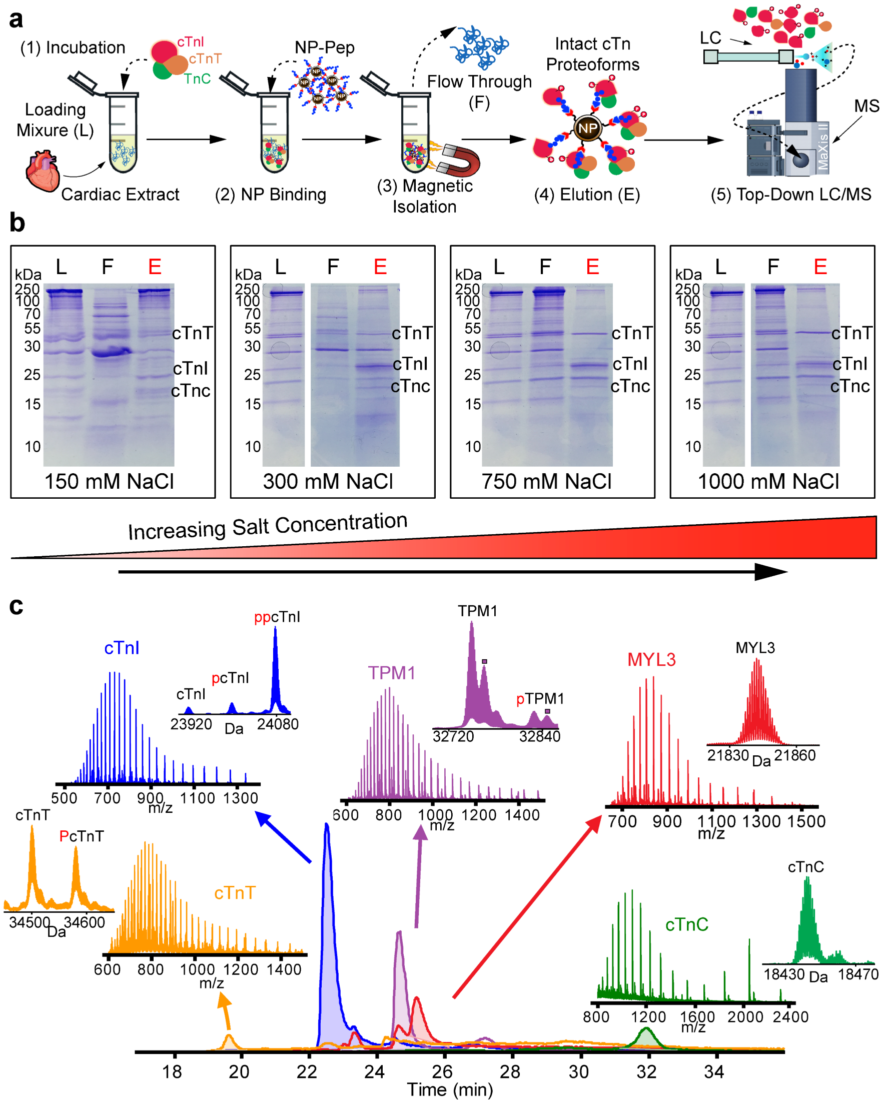

Supplementary Figure 13

Optimization of salt concentration in the wash buffer for effective cTnI enrichment.

a, Schematic illustration of the nanoproteomics strategy for enrichment of cTnI and its associating protein-protein interactors and top-down LC/MS analysis of proteins associated with cTnI. **b,** SDS-PAGE stained with Coomassie blue, visualizing that human cardiac sarcomere protein interacting partners of cTnI can be obtained during enrichment by simply tuning the salt concentration (150-1000 mM NaCl) of the wash buffer during the enrichment workflow. Equal amount (2 µg) of the loading mixture (L), flow-through (F), and elution mixture (E) after enrichment was loaded on the gel. **c,** Normalized extracted ion chromatograms and MS analysis of sarcomeric proteins obtained from the elution fraction after NP-Pep enrichment using 300 mM NaCl washing buffer. Deconvoluted MS for each protein is presented. 300 mM NaCl was ultimately used for all sarcomeric protein extract enrichment as it provided an optimal balance between cTnI enrichment specificity and overall cTnI recovery. TMP1 (α-tropomyosin), and MYL3 (myosin light chain 3) are named from the UniProtKB sequence database. Squares indicate +16 Da proteoform. p, phosphorylation; pp, bisphosphorylation.

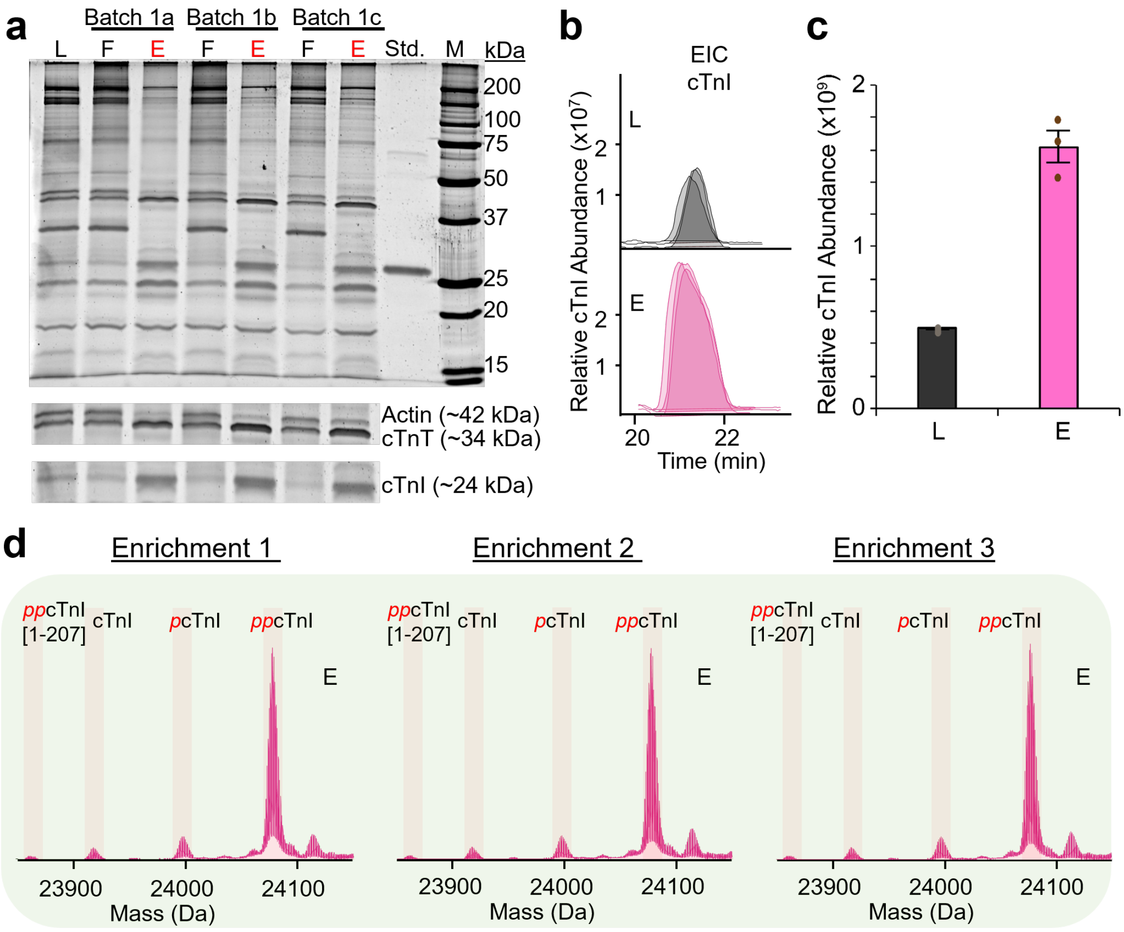

Supplementary Figure 14

High reproducibility of intra-batch enrichment by NP-Pep.

a, SDS-PAGE stained with SYPRO Ruby visualizing the cTnI enrichment performance and demonstrating the high reproducibility obtained from the same synthesis batch of Fe_3O_4-BAPTES NPs (intra-batch). Equal amounts of NP-Pep (5 mg) from the same synthesis batch were used for cTnI enrichment from sarcomeric protein extracts (300 µg) containing 0.3% cTnI obtained from a human donor heart. Equal amount (500 ng) of the loading mixture (L), flow-through (F), and elution mixture after enrichment (E) was loaded on the gel. Lower panels display enlarged SDS-PAGE strips highlighting NP enrichment of cTnI (~24 kDa) and cTnT (~34 kDa) from the sarcomeric protein loading mixture. **b**, Normalized extracted ion chromatograms (EIC) of cTnI from the sarcomeric protein loading mixture (L) and elution mixture (E). **c**, Relative abundance of cTnI from L and E when normalizing for 500 ng total protein loading, demonstrating significant enrichment of cTnI in E. **d**, Normalized deconvoluted mass spectra of cTnI present in the E, demonstrating consistency of cTnI enrichment across three enrichment replicates performed using NPs synthesized from a single batch (intra-batch). N-terminally acetylated cTnI proteoforms following Met exclusion were identified using accurate intact mass measurement, using the most abundant mass based on the amino acid sequence of entry name TNNI3_human from the UniProtKB sequence database. p, phosphorylation; pp, bisphosphorylation. Std., human cTnI protein standard; M., protein marker. Data are representative of n = 3 independent experiments and error bars indicate standard error of the mean.

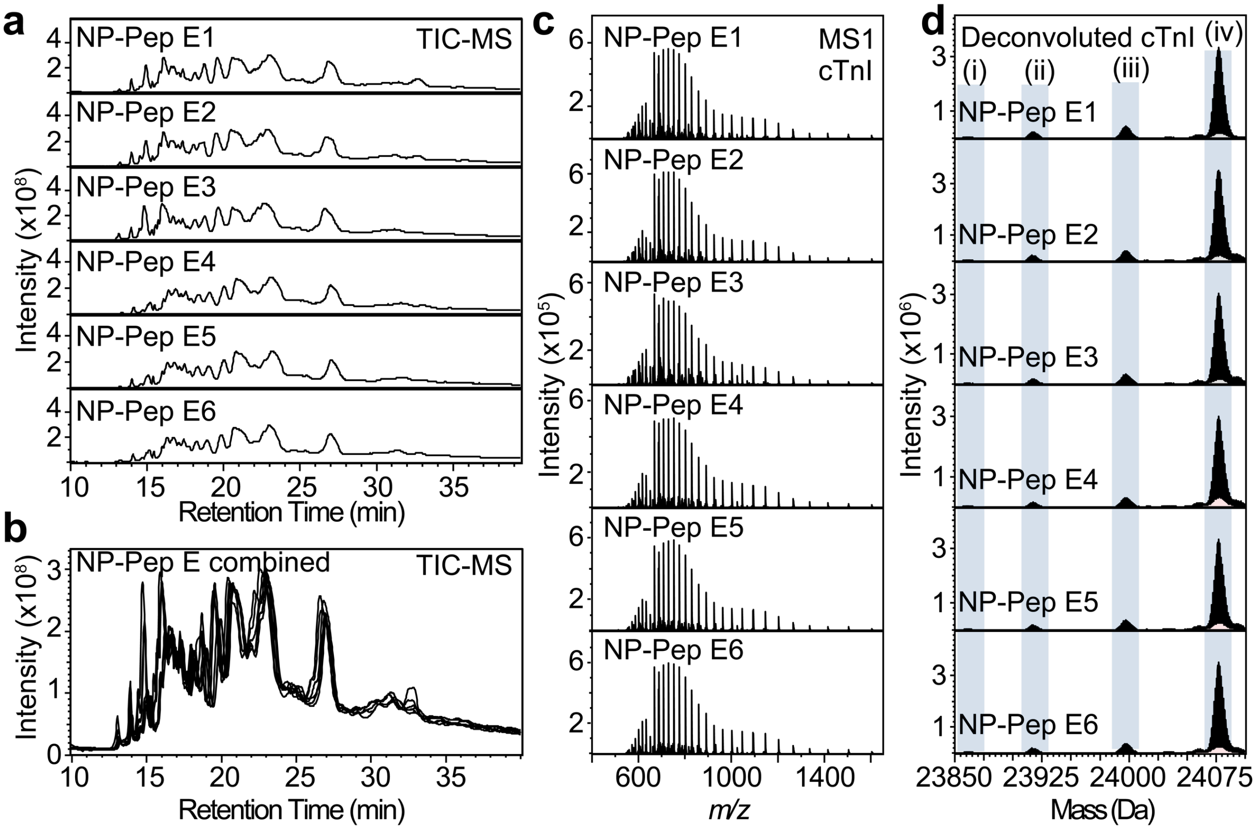

Supplementary Figure 15

Top-down LC/MS cTnI enrichment reproducibility by NP-Pep.

a-b, Total ion chromatogram mass spectra (TIC-MS) of six independent NP-Pep elution mixtures (E) obtained from human heart extracts. The elution mixtures are shown for each run (**a**) and overlaid in a single plot (**b**). Equal amounts of NP-Pep (5 mg) were used for cTnI enrichment containing 0.3% cTnI obtained from a human donor heart. **c**, Raw MS1 of cTnI obtained from the NP-Pep elution mixtures corresponding to (**a**-**b**). **d**, Deconvoluted mass spectra corresponding to enriched cTnI in (**a**-**c**). Roman numerals correspond to N-terminally acetylated cTnI proteoforms following Met exclusion: (i) ppcTnI[1-207]; (ii) cTnI; (iii) pcTnI; (iv) ppcTnI. cTnI proteoforms were identified using accurate intact mass measurement, using the most abundant mass based on the amino acid sequence of entry name TNNI3_human from the UniProtKB sequence database. Data correspond to the results shown in Fig. 2 and Supplementary Fig 14.

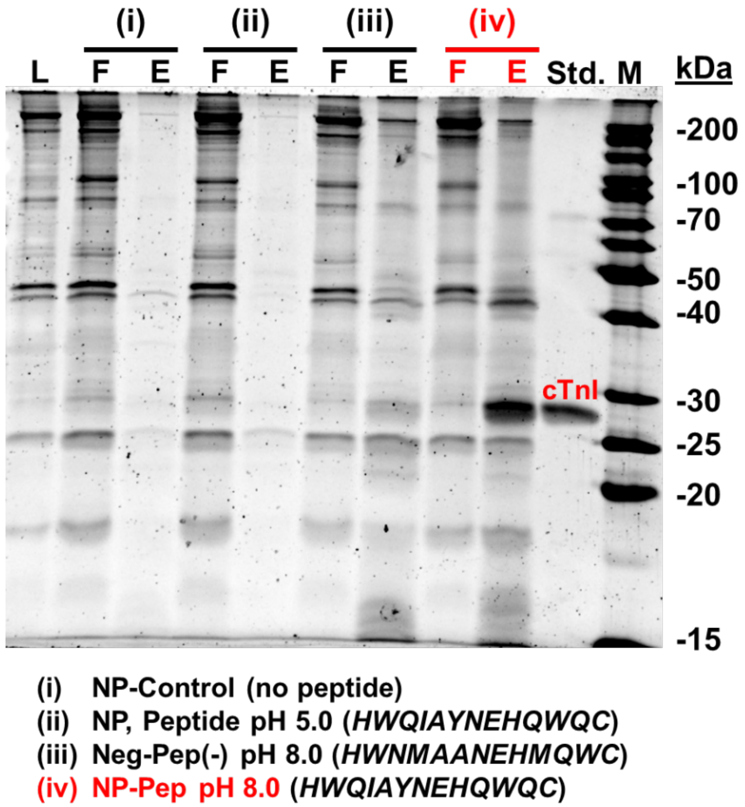

Supplementary Figure 16

Evaluation of the specificity of NP enrichment for cTnI using various control NPs.

Multiple NP controls (including unfunctionalized NPs or NPs functionalized sub-optimally) were compared against the NPs functionalized with the cTnI-binding peptide, demonstrating NPs functionalized with the correct cTnI-binding peptide (NP-Pep) are crucial for the successful enrichment. SDS-PAGE stained with SYPRO Ruby was used to compare the cTnI enrichment performance of i) unfunctionalized Fe_3O_4-BAPTES NPs with no peptide coupled (NP-Control); ii), Fe_3O_4-BAPTES NPs incubated with the high affinity cTnI peptide at pH 5.0 (NP, peptide pH 5.0); iii), Fe_3O_4-BAPTES NPs functionalized with a cTnI-negative control peptide (as shown in a previous report[6]) at pH 8.0 (Neg-Pep), and iv) the Fe_3O_4-BAPTES NPs functionalized with the high affinity cTnI peptide at pH 8.0 (NP-Pep). In (i), the unfunctionalized NPs do not appreciably enrich protein as seen in NP-control elution lane, demonstrating the resistance of the NPs to non-specific protein adsorption. In (ii), the reaction of the cysteine-thiol with BAPTES is inhibited at acidic pH (pH 5.0), leading to poor NP-peptide conjugation and downstream enrichment of cTnI. In (iii), the reaction of the cysteine-thiol with BAPTES occurs readily and specifically at slightly alkaline pH (pH 8.0), leading to NP-peptide conjugation. However, the use of a negative-binding sequence peptide with diminished binding affinity in (iii) results in poor cTnI enrichment. In (iv), the use of the high affinity cTnI binding peptide at slightly alkaline (pH 8.0) coupling conditions results in effective cTnI enrichment. cTnI is clearly enriched only in the NP-Pep elution lane, illustrating that proper surface chemistry and functionalization of the NP platform is critical for enabling cTnI enrichment. All NP samples were subjected to the same enrichment workflow. Equal amount (500 ng) of the loading mixture (L), flow-through (F), and elution mixture after enrichment (E) was loaded on the gel. 200 ng loading of endogenous human cTnI standard (Std.) was also loaded on the gel as a reference.

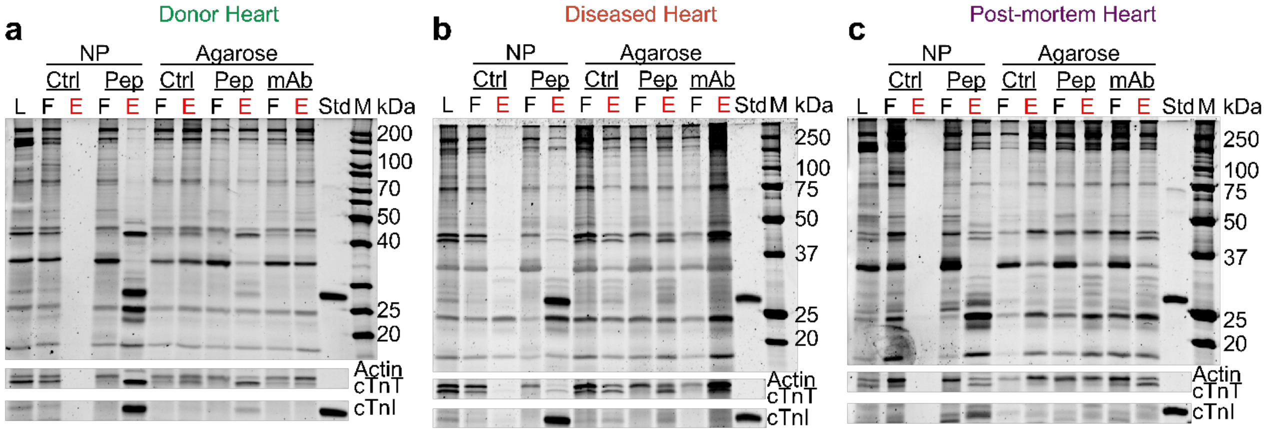

Supplementary Figure 17

Comparison of cTnI enrichment performance between the NP and Agarose platforms using sarcomeric protein mixtures extracted from a human donor heart, a diseased dilated cardiomyopathic heart, and a post-mortem heart extract.

a-c, SDS-PAGE stained with SYPRO Ruby visualizing the enrichment performance of the NP control (NP-Ctrl, no peptide), high affinity peptide-functionalized NP (NP-Pep), agarose-control (Agarose-Ctrl), high affinity peptide-functionalized agarose (Agarose-Pep), and antibody (mAb M46) functionalized agarose (Agarose-mAb). Sarcomeric protein extract (100 µg) obtained from a human donor heart (**a**), a diseased dilated cardiomyopathic heart (**b**), and a post-mortem heart (**c**) were used as the loading mixture, such that cTnI was not loaded in excess relative to the targeting ligands functionalized on the NPs or agarose. cTnI concentrations are listed in Supplementary Table 6. Lower panels display enlarged SDS-PAGE strips focusing on enrichment of cTnT and cTnI from (L). Equal amount (500 ng) of the loading mixture (L), flow-through (F), and elution mixture after enrichment (E) was loaded on the gel. The NP-Pep demonstrates the most effective enrichment across the different platforms examined here.

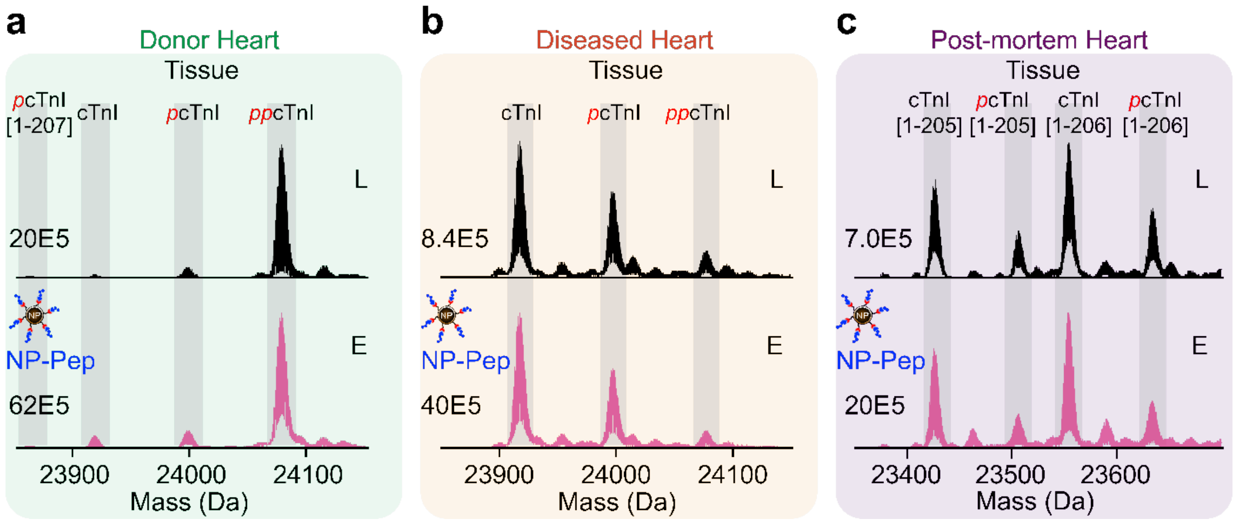

Supplementary Figure 18

Preservation of cTnI PTM profiles by the NP-Pep platform using sarcomeric protein extracts from a human donor heart, a dilated cardiomyopathic heart (diseased heart), and a post-mortem heart.

a-c, Deconvoluted mass spectra corresponding to reference cTnI proteoforms originating from a donor heart (**a**), a diseased dilated cardiomyopathic heart (**b**), and a post-mortem heart (**c**) before and after enrichment using the high affinity peptide-functionalized NP (NP-Pep). Equal amounts (500 ng) were loaded for LC/MS analysis. Data correspond to the same cTnI proteoform enrichment data presented in Fig. 2f-h, but are displayed at the same height to facilitate comparison (signal intensity is shown for each spectrum). Concentrations of cTnI used are listed in Supplementary Table 6. p, phosphorylation; pp, bisphosphorylation.

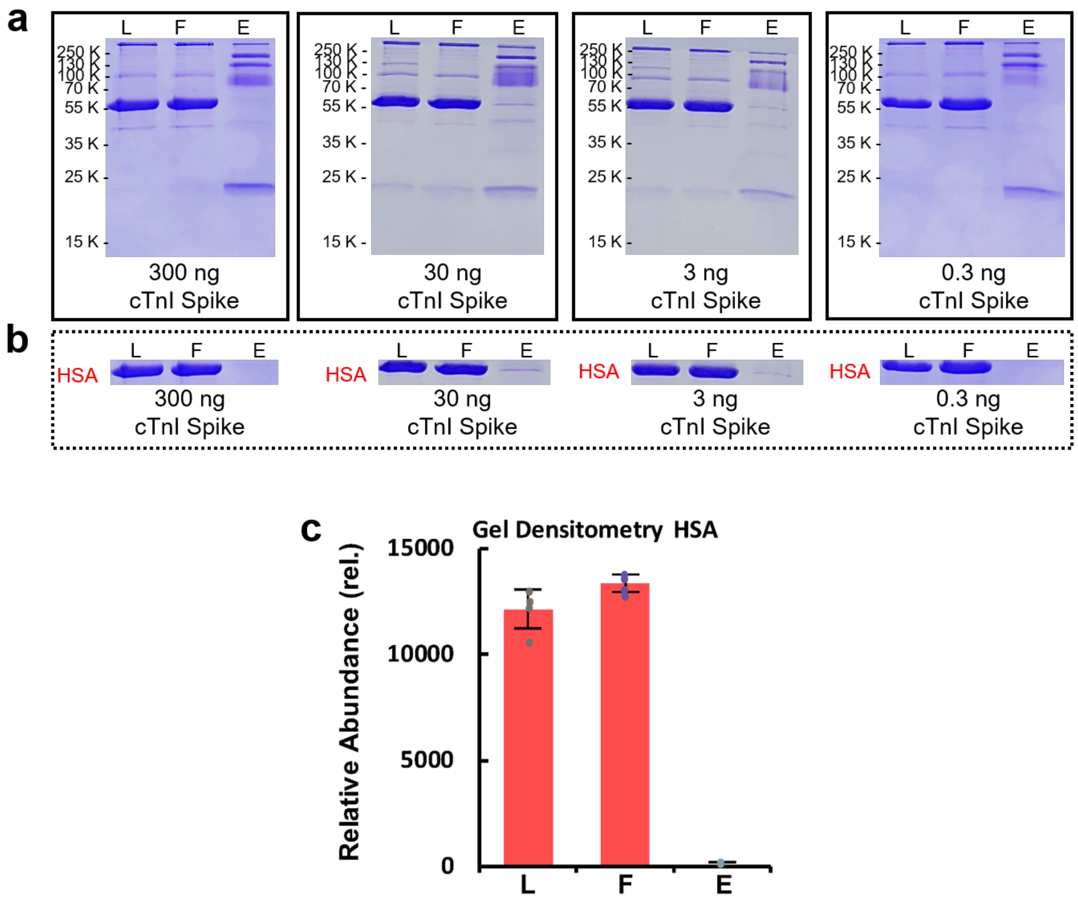

Supplementary Figure 19

Effective simultaneous depletion of human serum albumin (HSA) from protein mixture during NP-Pep enrichment of cTnI.

a, SDS-PAGE stained with Coomassie blue demonstrating highly effective simultaneous HSA depletion during cTnI enrichment using the NP platform from human serum (20 mg) at various spike-in cTnI concentrations (0.3 to 300 ng/mL). Equal amount (5 µg) of the loading mixture (L), flow-through (F), and elution mixture after enrichment (E) was loaded on the gel. **b**, Magnification of the HSA lane corresponding to (**a**). **c**, Gel densitometry relative quantitation of HSA depletion corresponding to the results shown in (**a**) and (**b**). HSA was depleted ~215-fold relative to the loading mixture following NP enrichment by ImageJ analysis. Data are representative of n = 4 independent experiments and error bars indicate standard deviation from the mean.

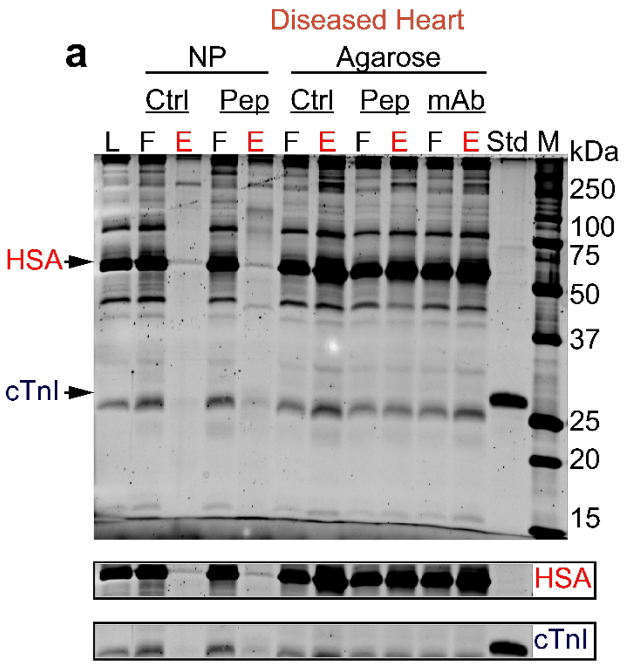

Supplementary Figure 20

Comparison of cTnI enrichment performance between the NP and Agarose platforms from human serum spiked with cTnI obtained from a diseased dilated cardiomyopathic heart and a post-mortem heart extract.

SDS-PAGE stained with SYPRO Ruby visualizing the enrichment performance of the NP control (NP-Ctrl, no peptide), high affinity peptide-functionalized NP (NP-Pep), agarose-control (Agarose-Ctrl), high affinity peptide-functionalized agarose (Agarose-Pep), and antibody (mAb M46) functionalized agarose (Agarose-mAb). Sarcomeric protein extract (100 µg) containing reference cTnI obtained from a diseased dilated cardiomyopathic heart (**a**) and a post-mortem heart (**b**) were spiked in human serum (10 mg) and used as the loading mixture, such that cTnI was not loaded in excess relative to the targeting ligands functionalized on the NPs or agarose. cTnI concentrations are listed in Supplementary Table 6. Lower panels display enlarged SDS-PAGE strips highlighting the depletion of HSA from the NP elution and the enrichment of cTnI. Equal amount (500 ng) of the loading mixture (L), flow-through (F), and elution mixture after enrichment (E) was loaded on the gel.

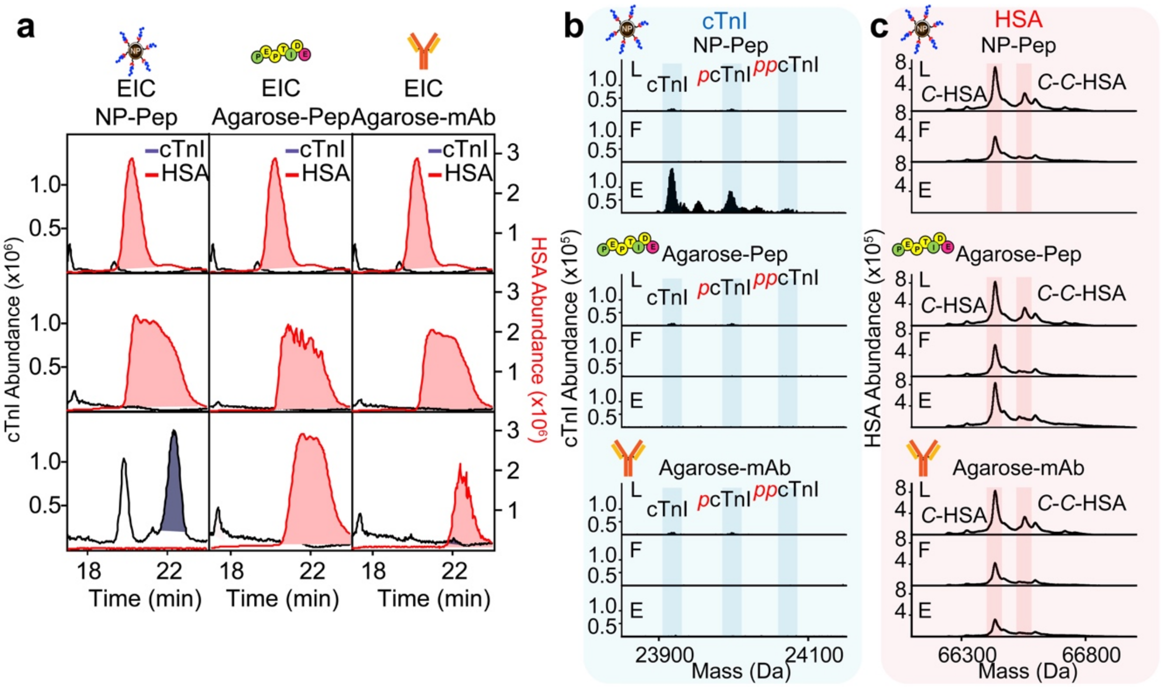

Supplementary Figure 21

LC/MS evaluation of cTnI enrichment from human serum with spike-in cTnI obtained from a human dilated cardiomyopathy heart extract, using the NP-Pep, Agarose-Pep, and Agarose-mAb.

a, Normalized extracted ion chromatograms (EICs) of cTnI (black) and HSA (red) corresponding to high affinity peptide-functionalized NP (NP-Pep), high affinity peptide-functionalized agarose (Agarose-Pep), and mAb-functionalized agarose (Agarose-mAb) enrichment from a human serum mixture with spike-in cTnI. cTnI (482 ng) from a human dilated cardiomyopathy heart extract was spiked into human serum (10 mg). **b**, Normalized deconvoluted mass spectra of cTnI obtained from the L, F, and E mixtures, corresponding to the data presented in (**a**). The high affinity peptide functionalized NPs (NP-Pep) show superior cTnI enrichment from human serum compared to the agarose functionalized with either the same high affinity peptide or a mAb targeting for the same epitope region as the peptide. **c**, Normalized deconvoluted mass spectra of HSA obtained from the L, F, and E mixtures and corresponding to the data presented in (**a**). The NP-Pep demonstrates superior depletion of the non-specific HSA to Agarose-Pep and Agarose-mAb. cTnI proteoforms (N-terminally acetylated following Met excision) were identified using accurate intact mass measurement, using the most abundant mass based on the amino acid sequence of entry name TNNI3_human from the UniProtKB sequence database. (L), loading mixture; (F), flow-through; (E), elution mixture; p*,* phosphorylation; pp*,* bisphosphorylation. *C*-HSA, cysteinylated human serum albumin; *C*-*C*-HSA, doubly-cysteinylated human serum albumin.

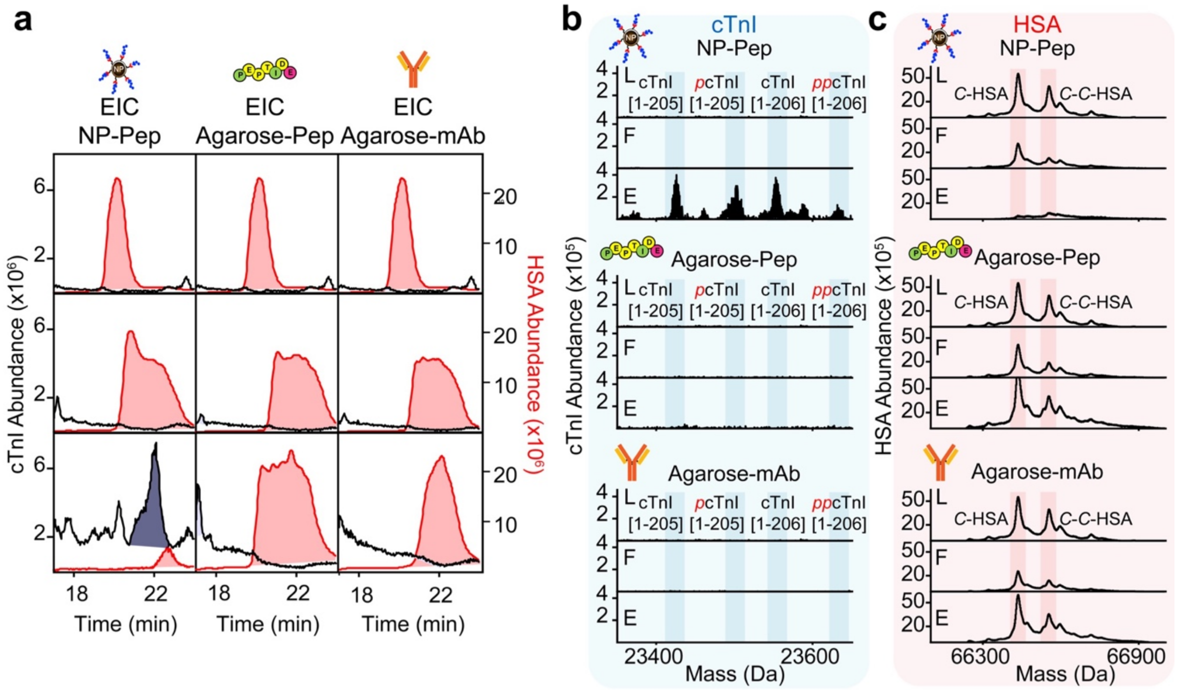

Supplementary Figure 22

LC/MS evaluation of cTnI enrichment from human serum with spike-in cTnI obtained from a post-mortem human heart extract, using the NP-Pep and Agarose-mAb platforms.

a, Normalized extracted ion chromatograms (EICs) of cTnI (black) and HSA (red) corresponding to high affinity peptide-functionalized NP (NP-Pep), high affinity peptide-functionalized agarose (Agarose-Pep), and mAb-functionalized agarose (Agarose-mAb) enrichment from a human serum mixture with spike-in cTnI. cTnI (465 ng) from a post-mortem heart extract was spiked into human serum (10 mg). **b**, Normalized deconvoluted mass spectra of cTnI obtained from the L, F, and E mixtures, corresponding to the data presented in (**a**). The high affinity peptide functionalized NPs (NP-Pep) show superior cTnI enrichment from human serum compared to the agarose functionalized with either the same high affinity peptide or a mAb targeting for the same epitope region as the peptide. **c**, Normalized deconvoluted mass spectra of HSA obtained from the L, F, and E mixtures and corresponding to the data presented in (**a**). The NP-Pep demonstrates superior resistance to non-specific HSA adsorption compared to Agarose-Pep and Agarose-mAb. cTnI proteoforms (N-terminally acetylated following Met excision) were identified using accurate intact mass measurement, using the most abundant mass based on the amino acid sequence of entry name TNNI3_human from the UniProtKB sequence database. (L), loading mixture; (F), flow-through; (E), elution mixture; p*,* phosphorylation; pp*,* bisphosphorylation. *C*-HSA, cysteinylated human serum albumin; *C*-*C*-HSA, doubly-cysteinylated human serum albumin.

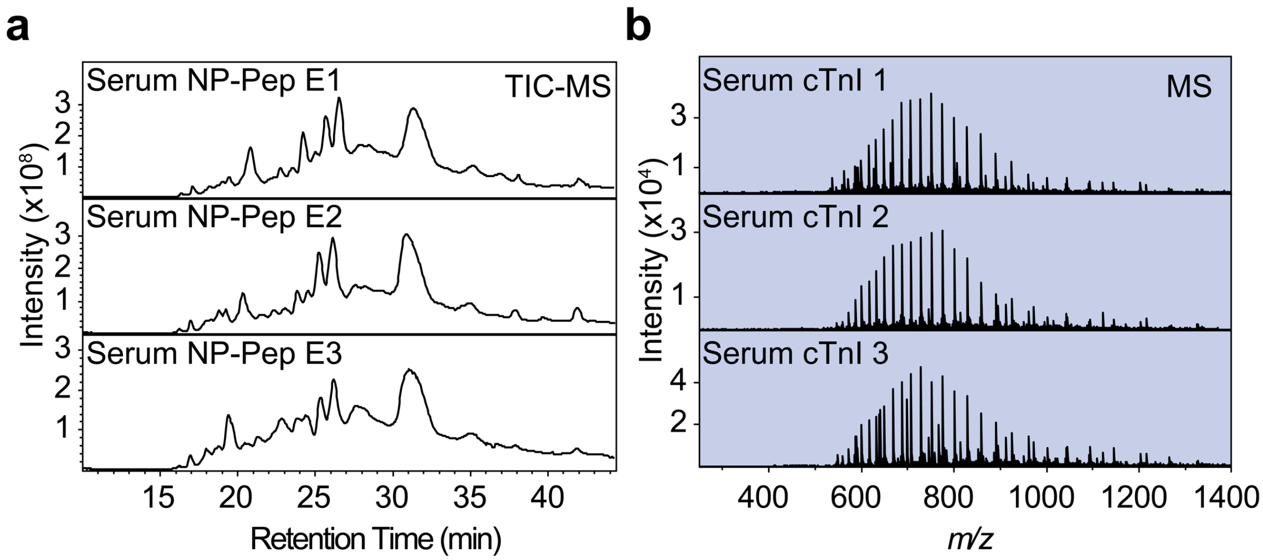

Supplementary Figure 23

Top-down LC/MS serum cTnI enrichment reproducibility and characterization by NP-Pep.
a, Total ion chromatogram mass spectra (TIC-MS) of three independent NP-Pep elution mixtures (E) obtained from a serum spike-in cTnI enrichment. Equal amounts of NP-Pep (5 mg) from separate synthesis batch were used for cTnI enrichment from human serum (10 mg) containing a minimal spike-in of cTnI (final concentration of 18.7 ng/mL), obtained from a human donor heart. **b**, Raw MS1 of cTnI obtained from the NP-Pep elution mixtures corresponding to (**a**).

Top 12 Most Frequently Identified Proteins Enriched from Serum

Gene Code	Protein Name	Molecular Weight (Da)	E-Value
TNNI3	Troponin I	24063.6894	3.78E-10
TNNC1	Troponin C	18498.5755	1.19E-18
LDB3	Isoform 6 of LIM domain-binding protein 3	30890.7825	2.88E-15
CRYAB	Alpha-crystallin B chain	20188.4295	7.99E-17
RS10	40S ribosomal protein S10	18941.921	1.02E-14
APOA1	Apolipoprotein A-I	11299.7765	1.68E-07
MYL3	Myosin light chain 3	21846.1696	5.07E-10
MLRV	Myosin regulatory light chain 2	18726.18	1.04E-09
H2B1D	Histone H2B type 1-D	10725.7183	9.55E-09
H2A2C	Histone H2A type 2-C	11000.1674	3.78E-15
APOE	Apolipoprotein E	34263.6597	6.71E-14
TNNT2	Isoform 11 of Troponin T	34573.645	1.42E-03

Identified in all n = 5 idependent serum enrichments
Identified in n = 4 idependent serum enrichments
Identified in n = 3 idependent serum enrichments

Supplementary Figure 24
Summary and illustration of proteins identified by NP-Pep following serum enrichment. Table summarizing the top 12 most frequently identified proteins enriched by the NP-Pep from serum cTnI spike-in enrichments obtained by combining n = 5 independent enrichments. cTnI (TNNI3) is confidently identified in all enrichment trials. Protein E-Value score is reported for each identification.

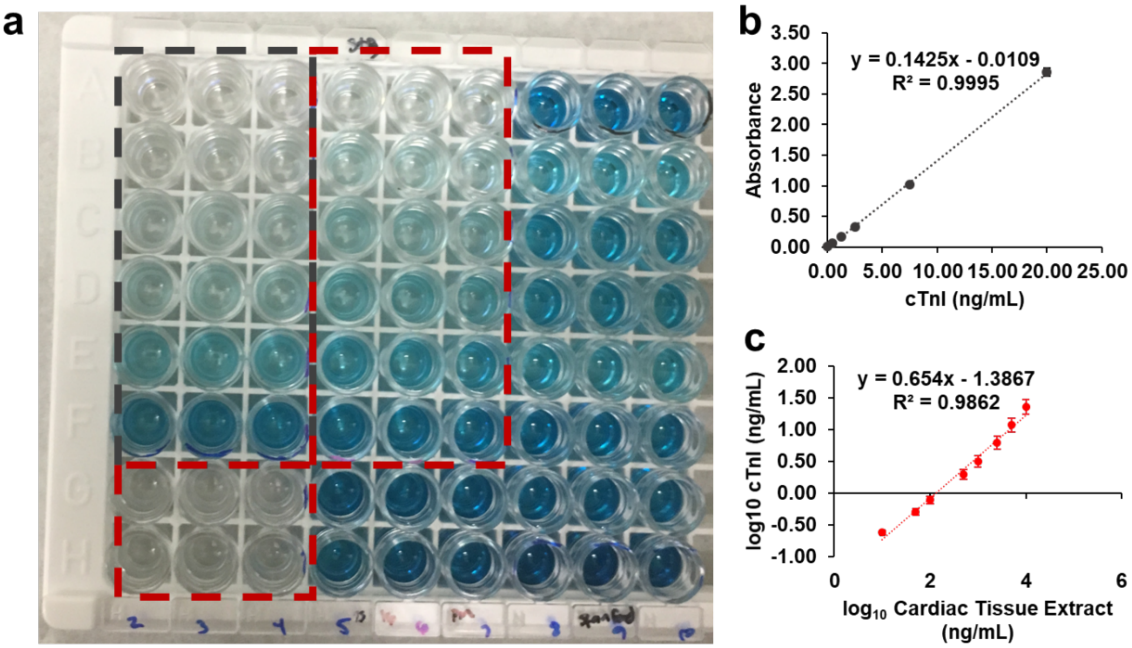

Supplementary Figure 25

ELISA-based cTnI quantification and Bradford total protein assay-based quantification of human serum with cTnI spike-in.

a, ELISA-based colorimetric quantification of cTnI standards (gray dashed box) and serum samples incrementally spike-in with sarcomeric protein extracts containing cTnI (red dashed boxes). All samples were dispensed in triplicate and the assay was performed according to the manufacturer's instructions. The ELISA assay uses capture antibodies targeting cTnI amino acids 18-28 and 86-90, with detection antibodies targeting cTnI amino acids 41-49. **b**, ELISA-based standard curve (0 ng, 0.4 ng, 1.25 ng, 2.5 ng, 7.5 ng, 20 ng) used for quantification of cTnI spike-in human serum, and quantification of MS-based sensitivity of cTnI detection. **c**, Log-log plot of cTnI concentration (ng/ml) quantified by ELISA, as a function of total sarcomeric protein spiked into serum (ng/ml) quantified by Bradford protein assay. Approximately 1 μg of sarcomeric protein extract contains 3 ng of cTnI. Data are representative of n = 3 independent experiments and error bars indicate standard deviation from the mean.

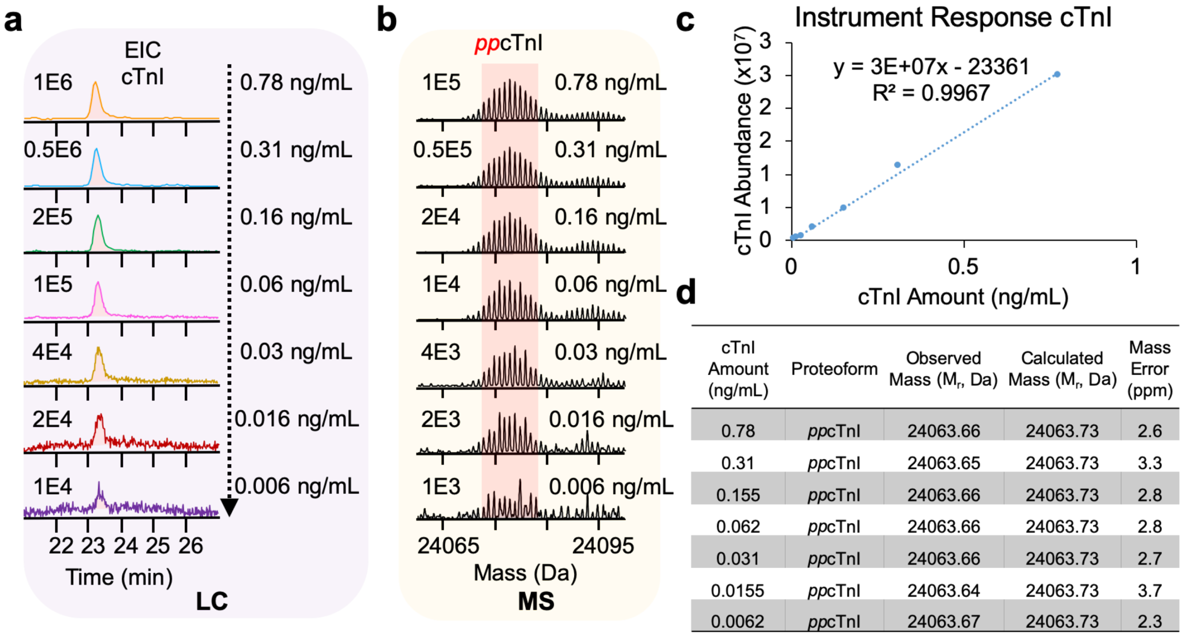

cTnI Amount (ng/mL)	Proteoform	Observed Mass (M_r, Da)	Calculated Mass (M_r, Da)	Mass Error (ppm)
0.78	*pp*cTnI	24063.66	24063.73	2.6
0.31	*pp*cTnI	24063.65	24063.73	3.3
0.155	*pp*cTnI	24063.66	24063.73	2.8
0.062	*pp*cTnI	24063.66	24063.73	2.8
0.031	*pp*cTnI	24063.66	24063.73	2.7
0.0155	*pp*cTnI	24063.64	24063.73	3.7
0.0062	*pp*cTnI	24063.67	24063.73	2.3

Supplementary Figure 26
Bruker MaXis II instrument limit of quantification (LOQ) and limit of detection (LOD) for cTnI.
a, Extracted ion chromatograms (EICs) for cTnI at various loading amounts (3.1-0.006 ng/mL) obtained from a heart tissue extract from a healthy donor heart tissue. Data correspond to the same data presented in Fig. 3e. **b**, Deconvoluted mass spectra corresponding to the same data presented in (**a**). ppcTnI is the most abundant proteoform of cTnI and is highlighted to illustrate the linear decrease in raw signal abundance as a function of concentration 3.1-0.006 ng/ml. **c**, Plot of relative cTnI abundance obtained from (**a**) at the various loadings tested, demonstrating linearity of MS-detection and response. From this plot, the LOD (3.3 σ/s) was determined to be 0.06 ng/ml and LOQ (10 σ/s) to be 0.2 ng/ml. **d**, Table summarizing the MS analysis of the data presented in (**a**-**c**). The mass accuracy (monoisotopic mass) at each tested cTnI loading is shown. All data are representative of n =2 independent experiments.

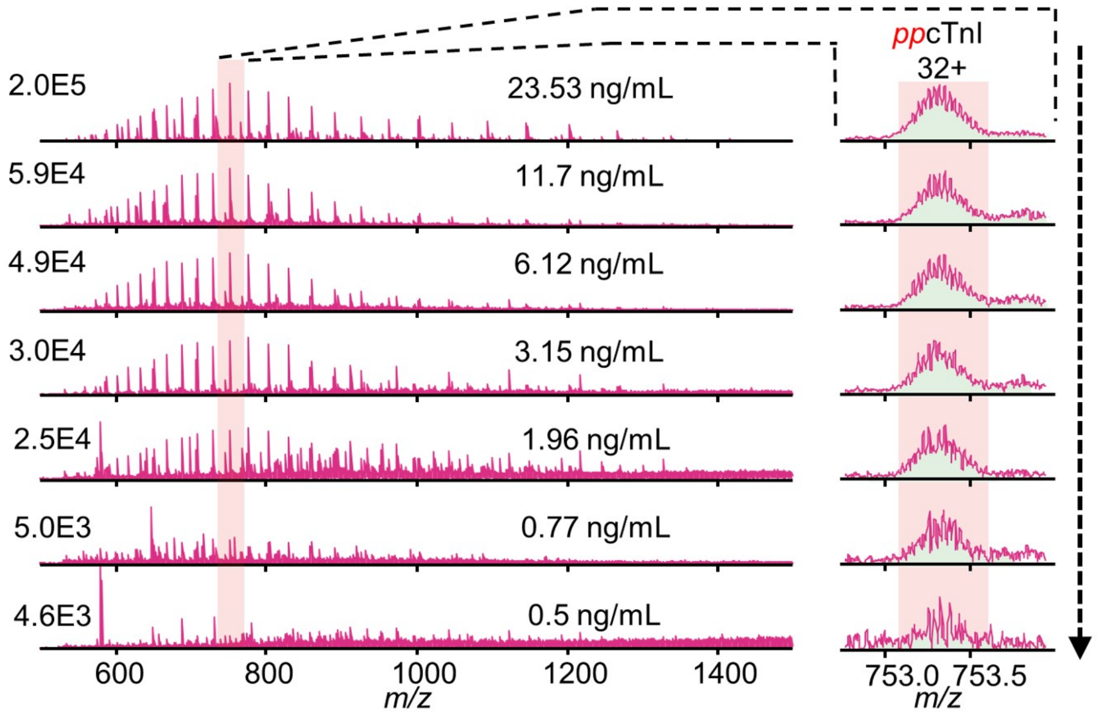

Supplementary Figure 27

Sensitivity of Top-Down MS-based cTnI assay using the nanoproteomics platform.

Normalized raw mass spectra corresponding to the same data presented in **Fig**. **3**. The most abundant signal, ppcTnI charge state 32+, is highlighted to illustrate the raw signal abundance as a function of the concentration from 22.53 ng/ml to 0.50 ng/ml. pp*,* bisphosphorylation.

a b

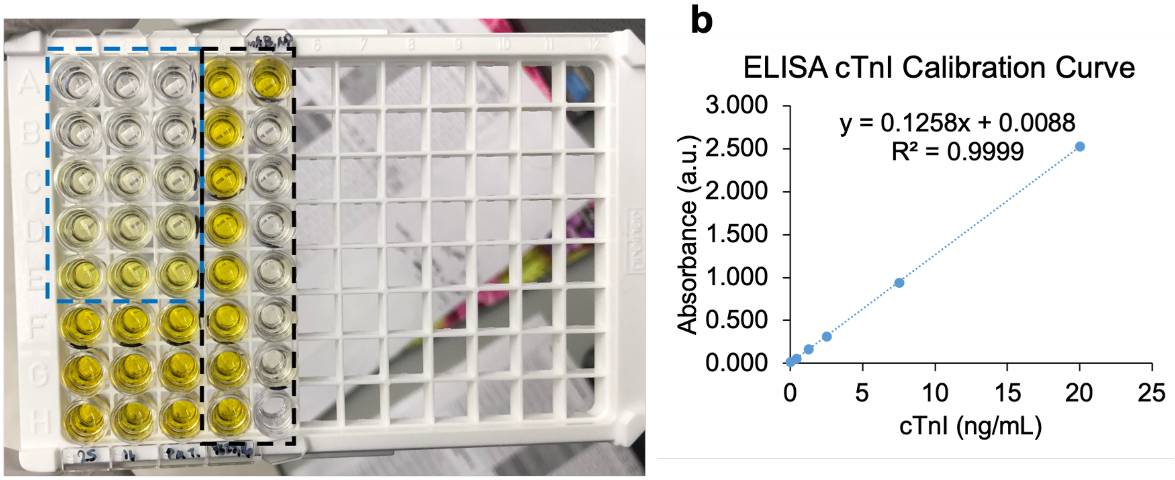

c

<table>
<tr><th>Sample</th><th>Sample Source</th><th>cTnI Enrichment Factor</th><th>Total cTnI Amount Before Enrichment (ng)</th><th>Total cTnI Amount Recovered After Enrichment (ng)</th><th>cTnI Percent Recovery (%)</th></tr>
<tr><td>NP-Pep</td><td>Tissue</td><td>1.7</td><td rowspan="3">302</td><td>268</td><td>89</td></tr>
<tr><td>NP-Pep</td><td>Serum</td><td>115</td><td>153</td><td>51</td></tr>
<tr><td>Agarose -mAb</td><td>Serum</td><td>39</td><td>53</td><td>17</td></tr>
</table>

Supplementary Figure 28
ELISA-based cTnI enrichment efficiency quantification of NP-Pep and Agarose-mAb.
a, ELISA-based colorimetric quantification of cTnI standards (blue dashed box) and enrichment samples (black dashed box). All samples were dispensed in triplicate and the assay was performed according to the manufacturer's instructions. The ELISA assay uses capture antibodies targeting cTnI amino acids 18-28 and 86-90, with detection antibodies targeting cTnI amino acids 41-49. **b**, ELISA-based standard curve (0 ng, 0.4 ng, 1.25 ng, 2.5 ng, 7.5 ng, 20 ng) used for quantification of cTnI amount before enrichment and after enrichment by NP-Pep or Agarose-mAb. **c**, Summary of enrichment performance results for NP-Pep (tissue/serum) and Agarose-mAb (serum).

$$\text{cTnI enrichment factor} = \frac{\text{concentration cTnI After Enrichment } (\frac{\text{ng}}{\text{mL}})}{\text{concentration cTnI Before Enrichment } (\frac{\text{ng}}{\text{mL}})}.$$

$$\text{cTnI percent recovery} = \frac{\text{cTnI After Enrichment (ng)}}{\text{cTnI Before Enrichment (ng)}} \times 100\%.$$

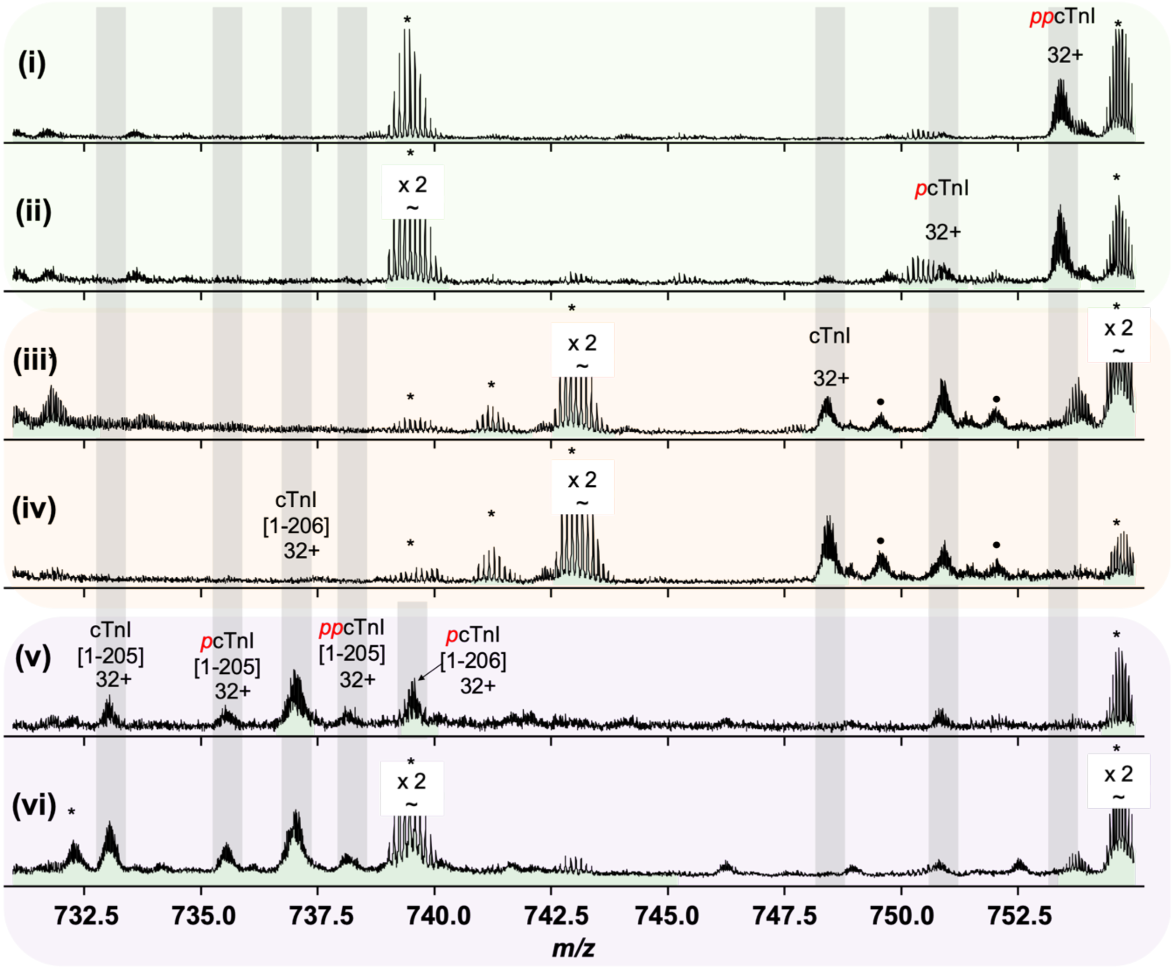

Supplementary Figure 29
LC/MS analysis of cTnI proteoforms enriched directly from human serum by NP-Pep.
Normalized raw mass spectra data corresponding to deconvoluted mass spectra shown in s The cTnI (~10-20 ng/mL) spiked in the human serum (10 mg) are extracted from various human hearts: (i) and (ii), donor hearts; (iii) and (iv), diseased hearts with dilated cardiomyopathy, (v) and (vi), post-mortem hearts. p, phosphorylation. pp, bisphosphorylation. Data are representative of n = 3 independent experiments. Asterisks "*" indicate co-eluting serum proteins and circles "•" indicate other cTnI proteoforms. A summary of the enriched proteoforms from serum and their respective mass measurements by top-down MS are listed in Supplementary Table 8.

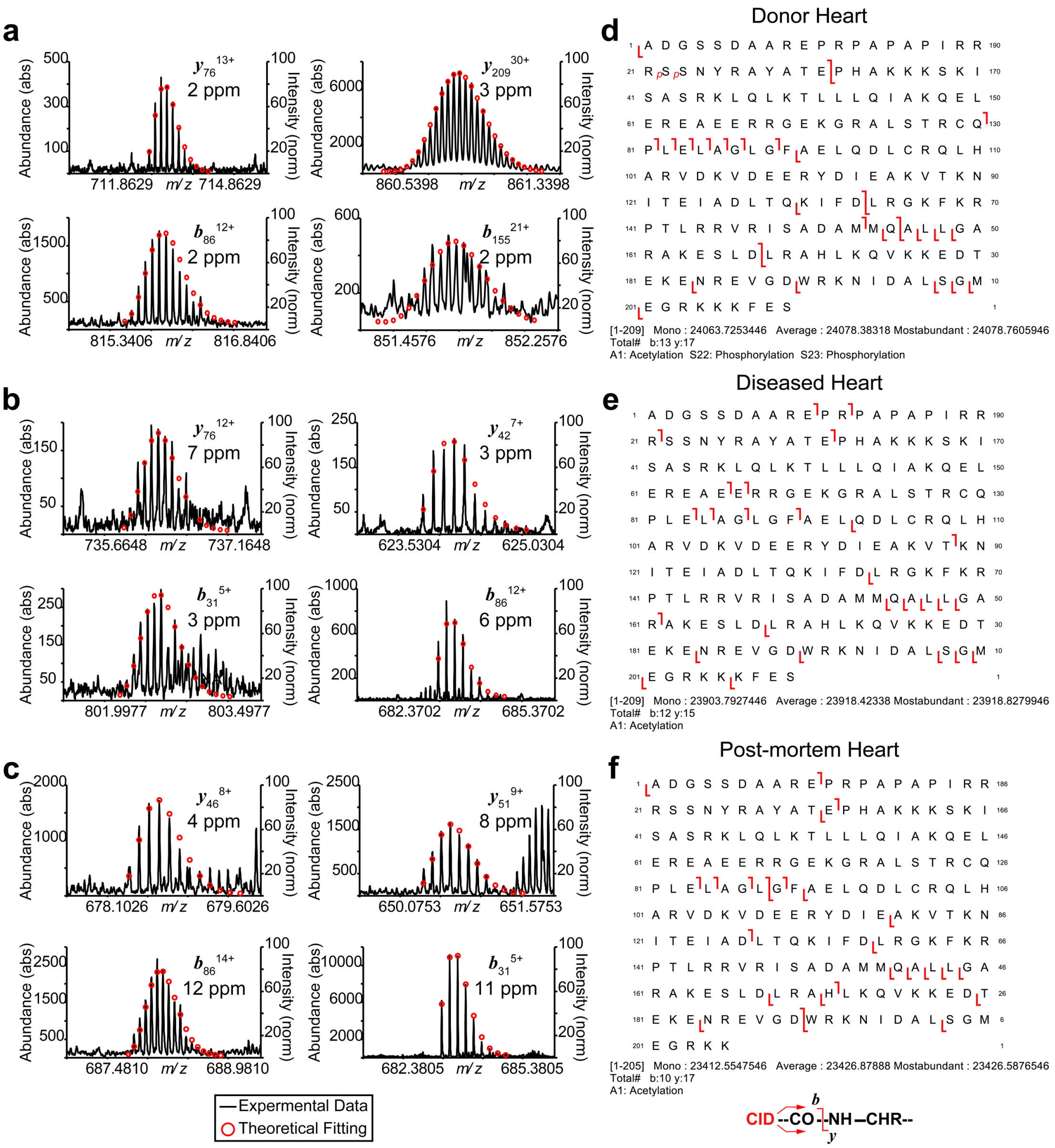

Supplementary Figure 30

Top-down LC-MS/MS characterization of cTnI arising from the various biological samples after enrichment.

a-c, Representative CID fragment ions obtained from cTnI arising from the donor heart (y_{76}^{13+}, y_{209}^{30+}, b_{86}^{12+}, and b_{155}^{21+}), the diseased heart (y_{76}^{12+}, y_{42}^{7+}, b_{31}^{5+}, and b_{86}^{12+}), and the post-mortem heart (y_{76}^{8+}, y_{51}^{9+}, b_{86}^{14+}, and b_{31}^{5+}) sources. cTnI was found to be primarily in its bisphosphorylated state in the donor heart (ppcTnI; Ser22, and Ser23), unphosphorylated state in the diseased heart (cTnI), and in a proteolytically degraded form in the post-mortem heart (cTnI[1-206]). Theoretical ion distributions are indicated by the red dots and mass accuracy errors are listed for each fragment ion. **d-f**, Protein sequence fragmentation mapping of the specific proteoform of cTnI corresponding to the fragment ion data obtained from each cTnI source (**a-c**). CID fragmentations are shown as red cleavages. All matched sequences contained N-terminally acetylated cTnI proteoforms following Met exclusion. Amino acid sequence was based on the entry name TNNI3_human obtained from the UniProtKB sequence database.

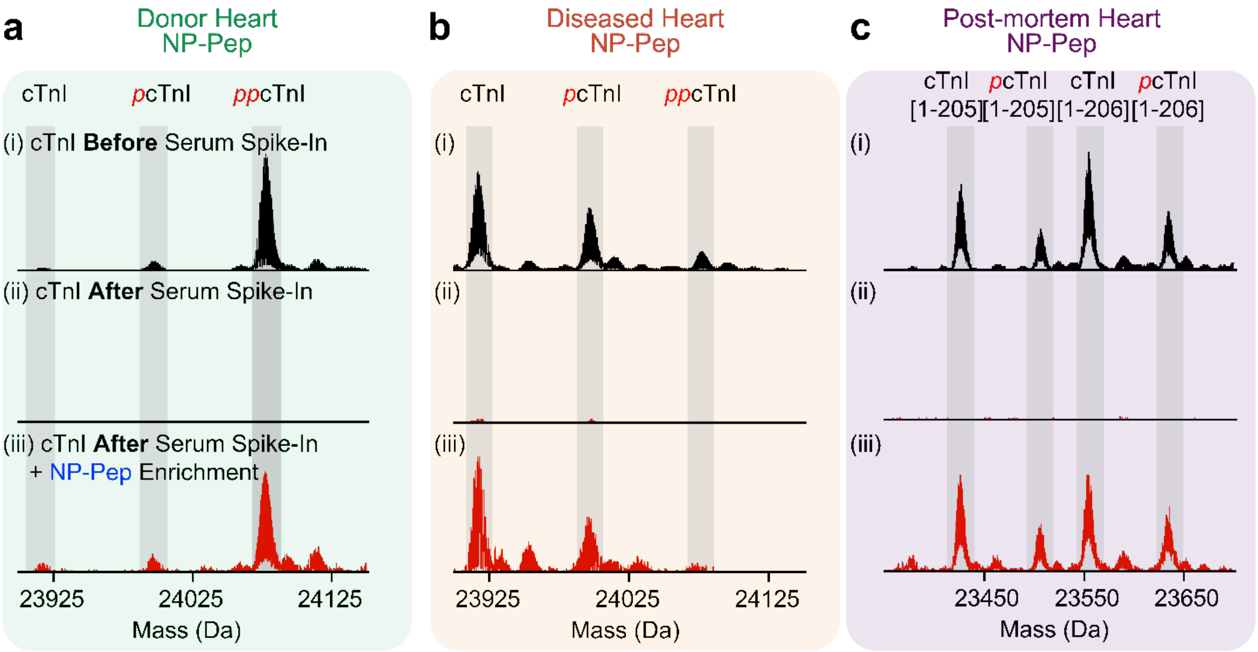

Supplementary Figure 31

Preservation of cTnI PTM profiles by NP-Pep from serum reflecting different cardiac pathophysiologies.

a-c, Representative deconvoluted mass spectra corresponding to cTnI proteoforms originating from a donor heart (**a**), a diseased dilated cardiomyopathic heart (**b**), and a post-mortem heart (**c**) before serum spike-in (i), after serum spike-in (ii), and after NP-Pep enrichment of human serum spiked with cTnI (iii). Equal amounts of NP-Pep (5 mg) were used for the cTnI enrichments and equal amounts (500 ng) were loaded for LC/MS analysis. Data correspond to the same cTnI proteoform data presented in Fig. 2 and Fig. 4 (Samples (i), (iv), and (v)). Concentrations of cTnI used are listed in Supplementary Table 6 and Supplementary Table 8. p, phosphorylation; pp, bisphosphorylation.

Supplementary Tables

Supplementary Table 1. Summary of various materials characterization and surface functionalization analysis of various functionalized Fe_3O_4 NPs synthesized at a large-scale (120 mg).

Particle	TEM Diameter (nm)	TGA Weight Loss (%)	Surface Ligand Coverage (ligands/nm^2)	Surface Ligand Relative Concentration (µmol ligand/mg NP)
Fe_3O_4-OA	8.03 ± 0.08	44	12	1.6
Fe_3O_4-BAPTES	8.2 ± 0.2	26	5	0.90
Fe_3O_4-Peptide	8.3 ± 0.2	32	0.2	0.034

All errors indicate standard deviations from measurements on three independent batches of particles.

Supplementary Table 2. Summary showing the details of using TGA analysis to estimate the surface density of BAPTES ligands on the surface of NPs.

**Nanoparticle**	**NP-BAPTES**
Average radius (obtained from TEM), *r*	4.0 nm
The mass of BAPTES ligand molecules (quantified by TGA % mass loss), *m*	26% × 4.313 mg (Sample size used for TGA) = 1.121 mg BAPTES
Total number of BAPTES ligand molecules = $\frac{m}{\mathrm{MW}} \times \mathrm{N_A}$ where MW, ligand molecular weight; $\mathrm{N_A}$, Avogadro's number ($6.022 \times 10^{20}\ \frac{\text{molecules}}{\text{mmol}}$)	$\frac{1.121\ \text{mg BAPTES}}{287.12\ \frac{\text{mg}}{\text{mmol}}} \times 6.022 \times 10^{20}\ \frac{\text{molecules}}{\text{mmol}}$ = ~ 2.35×10^{18} BAPTES ligand molecules
The total mass (*M*) of NPs in TGA sample	$4.313\ \text{mg sample} - 1.121\ \text{mg BAPTES} = 3.192\ \text{mg NPs}$
The total volume (*V*) of NPs from TGA sample $\left(\rho = \frac{5.18\ \text{g}}{\text{cm}^3}\right)$	$V = M / \rho$; $6.162 \times 10^{-4}\ \text{cm}^3$
The volume (*v*) of a single NP	$v = \frac{4}{3}\pi\,(4 \times 10^{-7}\text{cm})^3 = 2.68 \times 10^{-19}\text{cm}^3$
Total # of NPs from TGA sample, N:	$\frac{V}{v} = \frac{6.95 \times 10^{-4}\ \text{cm}^3}{2.68 \times 10^{-19}\text{cm}^3} = 2.298 \times 10^{15}$ NPs
# of BAPTES ligands per NP	~1023
SA (total surface area) N × $4\pi r^2$	$4.622 \times 10^{17}\ \text{nm}^2$
# of BAPTES ligands per nm^2	~5
μmol BAPTES ligands per mg of nanoparticle	~0.90 μmol BAPTES/mg of NP

Supplementary Table 3. Summary showing the details of using TGA analysis TGA analysis to estimate the surface density of cTnI peptide ligands on the surface of NPs.

**Nanoparticle**	**NP-Pep**
Average radius (obtained from TEM), *r*	4.0 nm
The mass of BAPTES and peptide ligand molecules (quantified by total TGA % mass loss)	32% × 2.434 mg (Sample size used for TGA) = 0.779 mg BAPTES + Peptide ligand
The mass of peptide ligand molecules (quantified by TGA % mass loss (i.e. 26%) compared to NP-BAPTES), *m*	6 % × 2.434 mg (Sample size used for TGA) = 0.146 mg peptide
Total number of peptide ligand molecules = $\frac{m}{\mathrm{MW}} \times \mathrm{N_A}$ where MW, ligand molecular weight; $\mathrm{N_A}$, Avogadro's number ($6.022 \times 10^{20} \frac{\text{molecules}}{\text{mmol}}$)	$\frac{0.146\ \text{mg peptide}}{1742.89\ \frac{\text{mg}}{\text{mmol}}} \times 6.022 \times 10^{20}\ \frac{\text{molecules}}{\text{mmol}}$ = ~ 5.046×10^{16} peptide molecules
The total mass (*M*) of NPs in TGA sample	2.434 mg sample $-$ 0.779 mg BAPTES $+$ peptide $=$ 1.655 mg NPs
The total volume (*V*) of NPs from TGA sample $\left(\rho = \frac{5.18\ \text{g}}{\text{cm}^3}\right)$	$V = M / \rho$; 3.195×10^{-4} cm^3
The volume (*v*) of a single NP	$v = \frac{4}{3}\pi\,(4 \times 10^{-7}\text{cm})^3 = 2.68 \times 10^{-19}\text{cm}^3$
Total # of NPs from TGA sample, N:	$\frac{V}{v} = \frac{3.197 \times 10^{-4}\ \text{cm}^3}{2.68 \times 10^{-19}\text{cm}^3} = 1.191 \times 10^{15}$ NPs
# of peptide ligands per NP	~42
SA (total surface area) N × $4\pi r^2$	2.396×10^{17} nm^2
# of peptide ligands per nm^2	~0.2
µmol peptide ligands per mg of nanoparticle	~0.034 µmol peptide/mg of NP

Supplementary Table 4. Summary of sarcomeric protein extraction buffers and reagents. All heart tissue extracts were prepared using the described extraction protocol.

**Wash Buffer (pH 7.0)**		
**Reagent (Stock concentration)**	**Final concentration**	**Purpose**
NaH_2PO_4 (500 mM)	5 mM	Maintain buffering capacity
Na_2HPO_4 (100 mM)	5 mM	Maintain buffering capacity
$MgCl_2$ (100 mM)	5 mM	Maintain ionic strength of medium
NaCl (137 mM)	100 mM	Maintain ionic strength of medium
EGTA (100 mM)	0.5 mM	Chelation of metal ions; inhibition of metalloproteases
**Add remaining reagents immediately prior to performing wash protocol**		
Triton™ X-100 (10% v/v)	1% (v/v)	Solubilization of poorly soluble proteins
DTT (1 M)	5 mM	Reducing agent
Halt™ protease inhibitor cocktail (100x)	1x	Inhibition of Ser/Cys/Asp proteases
PMSF (25 mM in ethanol)	1 mM	Inhibition of Ser proteases
Phosphatase inhibitor cocktail A (100x)	1x	Inhibition of Ser/Thr/Tyr phosphatases

**Protein Extraction Buffer (pH 7.5)**		
**Reagent (Stock concentration)**	**Final concentration**	**Purpose**
Tris (1 M)	25 mM	Maintain buffering capacity
$CaCl_2$ (100 mM)	0.1 mM	Maintain ionic strength of medium
LiCl (3.5 M)	700 mM	Protein extraction
EGTA (100 mM)	5 mM	Chelation of metal ions; inhibition of metalloproteases
**Add remaining reagents immediately prior to performing extraction protocol**		
DTT (1 M)	5 mM	Reducing agent
Halt™ protease inhibitor cocktail (100x)	1x	Inhibition of Ser/Cys/Asp proteases
PMSF (25 mM in ethanol)	1 mM	Inhibition of Ser proteases
Phosphatase inhibitor cocktail A (100x)	1x	Inhibition of Ser/Thr/Tyr phosphatases

Supplementary Table 5. Summary of accurate mass measurement by top-down MS analysis of NP-Peptide enriched cTnI proteoforms (i)-(iv) corresponding to the data presented in Fig. 2e. Most abundant masses are shown.

Sample	Proteoform	Observed Mass (Da)	Calculated Mass (Da)	Mass Error (ppm)
**Batch 1**	(i) ppcTnI[1-207]	23861.66	23861.68	0.8
	(ii) cTnI	23917.80	23917.83	1.3
	(iii) pcTnI	23997.78	23997.90	5.0
	(iv) ppcTnI	24077.73	24077.86	5.4
**Batch 2**	(i) ppcTnI[1-207]	23861.75	23861.68	2.9
	(ii) cTnI	23917.92	23917.83	3.8
	(iii) pcTnI	23997.89	23997.90	0.4
	(iv) ppcTnI	24077.85	24077.86	0.4
**Batch 3**	(i) ppcTnI[1-207]	23861.79	23861.68	4.6
	(ii) cTnI	23917.89	23917.83	2.5
	(iii) pcTnI	23997.89	23997.90	0.4
	(iv) ppcTnI	24077.85	24077.86	0.4

Supplementary Table 6. Summary of accurate mass measurement by top-down MS analysis of NP-Peptide enriched cTnI proteoforms (i)-(iv) corresponding to the data presented in Fig. 2f-h. Most abundant masses are shown.

Heart Sample	Proteoform	[cTnI]	Observed Mass (Da)	Calculated Mass (Da)	Mass Error (ppm)
**Donor**	(i) ppcTnI[1-207]	306 ng/ml	23861.73	23861.68	2.1
	(ii) cTnI		23917.87	23917.83	1.7
	(iii) pcTnI		23997.82	23997.90	3.3
	(iv) ppcTnI		24077.80	24077.86	2.5
**Dilated cardiomyopathy**	(ii) cTnI	482 ng/ml	23917.71	23917.83	5.0
	(iii) pcTnI		23997.77	23997.90	5.4
	(iv) ppcTnI		24077.73	24077.86	5.4
**Post-mortem**	(v) cTnI[1-205]	465 ng/ml	23426.67	23426.59	3.4
	(vi) pcTnI[1-205]		23506.65	23506.55	4.3
	(vii) cTnI[1-206]		23554.76	23554.68	3.4
	(viii) pcTnI[1-206]		23634.72	23634.65	3.0

Supplementary Table 7. Summary of the population characteristics of various non-diseased donor, diseased (dilated cardiomyopathy), and post-mortem heart samples corresponding to the data presented in Figure 4b and Supplementary Fig. 29.

Sample Code (Fig. 4b; Fig. S29)	Decoded ID	Specimen Type	Age	Sex	Ethnicity	ß-blocker?	Known medications
(i)	YG25	Donor Heart	61	M	--	--	--
(ii)	YG40	Donor Heart	25	F	--	--	Duonebs, Budesonide, Dexamethasone, Flovent, Sertraline, Mirena, Albuterol, Epipen
(iii)	YG13	Dilated Cardiomyopathy Heart	22	M	--	Yes	--
(iv)	YG16	Dilated Cardiomyopathy Heart	--	M	Caucasian	--	--
(v)	A08-089	Post-mortem Heart	58	F	Caucasian	Yes	aspirin, atenolol, budesonide, bupropion, calcium, cyancobalamin, duloxetine, ergocalciferol, ferrous sulfate, gabapentin, lamotrigine, levothyroxine, loratadine, methadone, modafinil, multivitamin, oxybutynin, pantoprazole, prednisone, piperacillin tazob
(vi)	A08-451	Post-mortem Heart	39	M	Caucasian	Yes	oxycodone, insulin, zocor, diflucan, macrobid, plavix, mycelex, aspirin, tums, colace, cardura, zetia, lasix, norco, prevacid, metoprolol, myfortic, lovaza, KCl, prednisone, flomax, valsartan, flexeril, lexapro, xanax, aranesp, ergocalciferol, nitroglycerin

Supplementary Table 8. Summary of accurate mass measurement by top-down MS analysis of NP-Peptide enriched cTnI proteoforms from human serum corresponding to the data presented in Fig. 4. Most abundant masses are shown.

Heart Sample	Proteoform	[cTnI]	Observed Mass (Da)	Calculated Mass (Da)	Mass Error (ppm)
**(i) Donor**	pcTnI	11.05 ng/ml	23997.70	23997.90	8.3
	ppcTnI		24077.64	24077.86	9.1
**(ii) Donor**	cTnI	10.69 ng/ml	23917.77	23917.83	2.5
	pcTnI		23997.70	23997.90	8.3
	ppcTnI		24077.64	24077.86	9.1
**(iii) Dilated cardiomyopathy**	cTnI	20.72 ng/ml	23917.77	23917.83	2.5
	pcTnI		23997.77	23997.90	5.4
**(iv) Dilated cardiomyopathy**	cTnI	22.84 ng/ml	23917.72	23917.83	4.6
	pcTnI		23997.77	23997.90	5.4
**(v) Post-mortem**	cTnI[1-205]	11.66 ng/ml	23426.40	23426.59	8.1
	pcTnI[1-205]		23506.37	23506.55	7.7
	cTnI[1-206]		23554.49	23554.68	8.1
	pcTnI[1-206]		23634.43	23634.65	9.3
**(vi) Post-mortem**	cTnI[1-205]	15.22 ng/ml	23426.56	23426.59	1.3
	pcTnI[1-205]		23506.52	23506.55	1.3
	cTnI[1-206]		23554.65	23554.68	1.3
	pcTnI[1-206]		23634.61	23634.65	1.7
